# War drives forest fire risks and highlights the need for more ecologically-sound forest management in post-war Ukraine

**DOI:** 10.1038/s41598-024-54811-5

**Published:** 2024-02-19

**Authors:** Maksym Matsala, Andrii Odruzhenko, Taras Hinchuk, Viktor Myroniuk, Igor Drobyshev, Serhii Sydorenko, Sergiy Zibtsev, Brian Milakovsky, Dmitry Schepaschenko, Florian Kraxner, Andrii Bilous

**Affiliations:** 1 Swedish University of Agricultural Sciences, Alnarp, Sweden; 2 National University of Life and Environmental Sciences of Ukraine, Kyiv, Ukraine; 3 Ukrainian Hydrometeorological Institute, Kyiv, Ukraine; 4 Ukrainian Order ‘Sign of Honor’ Research Institute of Forestry and Forest Melioration Named After G. M. Vysotsky, Kharkiv, Ukraine; 5 Jurmala, Latvia; 6 International Institute for Applied Systems Analysis, Laxenburg, Austria; 7 University of Quebec at Abitibi-Temiscamingue, Ville-Marie, Canada

**Keywords:** Environmental impact, Forestry

## Abstract

Since 24 February 2022, Ukraine has experienced full-scale military aggression initiated by the Russian Federation. The war has had a major negative impact on vegetation cover of war-affected regions. We explored interactions between pre-war forest management and the impacts of military activities in three of the most forested Ukrainian areas of interest (AOI), affected by the war. These were forests lying between Kharkiv and Luhansk cities (AOI ‘East’), forests along the Dnipro River delta (AOI ‘Kherson’), and those of the Chornobyl Exclusion Zone (AOI CEZ). We used Sentinel satellite imagery to create damaged forest cover masks for the year 2022. We mapped forests with elevated fire hazard, which was defined as a degree of exposure to the fire-supporting land use (mostly an agricultural land, a common source of ignitions in Ukraine). We evaluated the forest disturbance rate in 2022, as compared to pre-war rates. We documented significant increases in non-stand replacing disturbances (low severity fires and non-fire disturbances) for all three of the AOIs. Damaged forest cover varied among the AOIs (24,180 ± 4,715 ha, or 9.3% ± 1.8% in the ‘East’ AOI; 7,293 ± 1,925 ha, or 15.7% ± 4.1% in the ‘Kherson’ AOI; 7,116 ± 1,274 ha, or 5.0% ± 0.9% in the CEZ AOI). Among the forests damaged in 2022, the ‘Kherson’ AOI will likely have the highest proportion of an area with elevated fire hazard in the coming decades, as compared to other regions (89% vs. 70% in the ‘East’ and CEZ AOIs respectively). Future fire risks and extensive war-related disturbance of forest cover call for forest management to develop strategies explicitly addressing these factors.

## Introduction

The Russian Federation initiated a full-scale military invasion of Ukraine on 24 February 2022. Over the next year and a half, a vast area in the southern and eastern parts of Ukraine came under massive artillery shelling and has been impacted by a variety of war-related activities^1^. The war has led to contamination of agricultural and forest landscapes by unexploded ordnance (UXO), wildland fires, and environmental pollution^2,3^. Forest fires due to shelling-related ignition likely was the most frequent cause of forest disturbance in the conflict-affected area. Their impact has been particularly severe as fire suppression operations have been challenging due to fires happening largely in isolated rural areas, ongoing battles, and UXO contamination^4^. The scale of environmental damage to Ukrainian territory, caused by the Russian aggression, is massive, although its full extent currently remains unknown due to the ongoing war.

The military activities and associated wildfires heavily compromised the ecosystem functions of the initially modest forest areas in the war-affected regions of Ukraine. These forests lay in a landscape matrix of croplands and industrialized zones and play a key role in sustaining landscape biodiversity and human well-being^5^. Forests in war-affected regions have been crucial for the protection of soils and regulation of water balance within respective watersheds^6^. These forests provided timber, firewood, and food resources for the local population. The state of these forests prior to the war and the legacy of their recent management might have increased their vulnerability to war-related disturbances^7^. In particular, the even-aged monocultures of Scots pine (*Pinus sylvestris* L.) that dominate in these areas have shown elevated levels of tree mortality and an increased risk of wildfires^8^. Over the last three decades, the total forest area along the Siverskyi Donets River (eastern part of Ukraine) and in Khersonska oblast (southern part of Ukraine) have declined and have become more fragmented (e.g.,^9^).

During the pre-war era (until 2014) and even thereafter, establishment of high-density Scots pine plantations remained at the core of forest regeneration programs in eastern and southern Ukraine. This development took place despite sensitivity of such plantations to drought, fires and growth decline due to limited soil moisture^10^. This practice has led to an increase in the forest fire hazard, which is further augmented by the lack of proactive fire management policies. The work to prevent uncontrolled forest fires in Ukraine has been largely limited to creating firebreaks and does not yet include fuel reduction treatments or prescribed burning^6^.

Abundance of fuels in pine plantations make forests susceptible to human-related ignitions originated on the agricultural land. Farmers use spring fires to remove weed and autumn fires to eliminate crop residues^11^, despite the fact that such fires are illegal in Ukraine. This practice has resulted in large fires in the past, as fires in the CEZ AOI and Luhanska oblast (both occurred in 2020), reach the size of 30,000 ha^12,13^. Recent studies have called for revision of the outdated forest management practices in fire prone landscapes of eastern and southern Ukraine^6,9^. Restoration of damaged forests following liberation of Ukraine may need to follow updated management policies, which will incorporate proactive management of fire risks. Shifting the focus from commonly dense and highly flammable coniferous monocultures to more structurally diverse and less flammable forests could be a crucial element of this transition.

Satellite imagery relying on spectral signatures of forested areas provides both fast and accurate evaluation of environmental damage to the ecosystems^14,15^. For example, Sentinel-1 (microwave, or radar) and Sentinel-2 (optical) satellites provide publicly available, frequent (5–6 days interval) and well-resolved (10–20 m resolution, multiple bands) data. Their capacity to detect and map both wildfires and tree harvest is well documented^16,17^. Satellite data sources help to assess the war-related land use change^7,18^ and can provide sufficiently long-term data on forest disturbance regimes (e.g. Landsat-based products^19,20^).

This study estimated war-related forest damage in three areas of interest (AOI) in Ukraine between April and September 2022, using publicly available Sentinel data. These AOIs were the CEZ AOI in the north of Ukraine (Kyivska oblast), the forested part of Khersonska oblast in the south, and forests along Siverskyi Donets River in the east of Ukraine (Kharkivska, Donetska and Luhanska oblasts). We hypothesized (H1) that the Russian invasion in 2022 led to significant change in the disturbed area in the ‘Kherson’ AOI but not in the CEZ and ‘East’ AOIs. This hypothesis was based on the fact that the ‘Kherson’ AOI did not exhibit any major disturbances since 2007. In contrast, the ‘East’ and CEZ AOIs are well-known for both frequent severe fires and continuous tree harvest or bark beetle outbreaks, respectively. To add in this analysis, we also used Landsat time series to develop the chronology of forest damages due to specific disturbance factors since 1986.

Fuel type and abundance likely controlled fires in war-affected forests. Harvesting operations and frequent fires in the ‘East’ and CEZ AOIs prior to invasion in 2022 might reduce fuel loads that limited extent of fires in that year. We hypothesized that higher levels of fire activity and harvest prior to 2022 corresponded to lower level of fire activity during the war in three studied AOIs (H2). To test H2, we compared proportions of burned forests in three studied AOIs.

Finally, to initiate discussion on the management of the damaged forests, we mapped their susceptibility to future fires^21^. To this end, we regarded the presence of the interface between war-damaged forest and open landscape as an indication of an elevated fire hazard. We based this assumption on the wide-spread practice of spring and autumn burnings of agricultural and other open lands in Ukraine^9^. We carried out landscape analysis to illustrate what proportion of damaged forests has an elevated fire hazard due to having common edges with open landscapes, which may facilitate rapid fire spread.

## Methods and materials

### Study area

**Figure 1:**
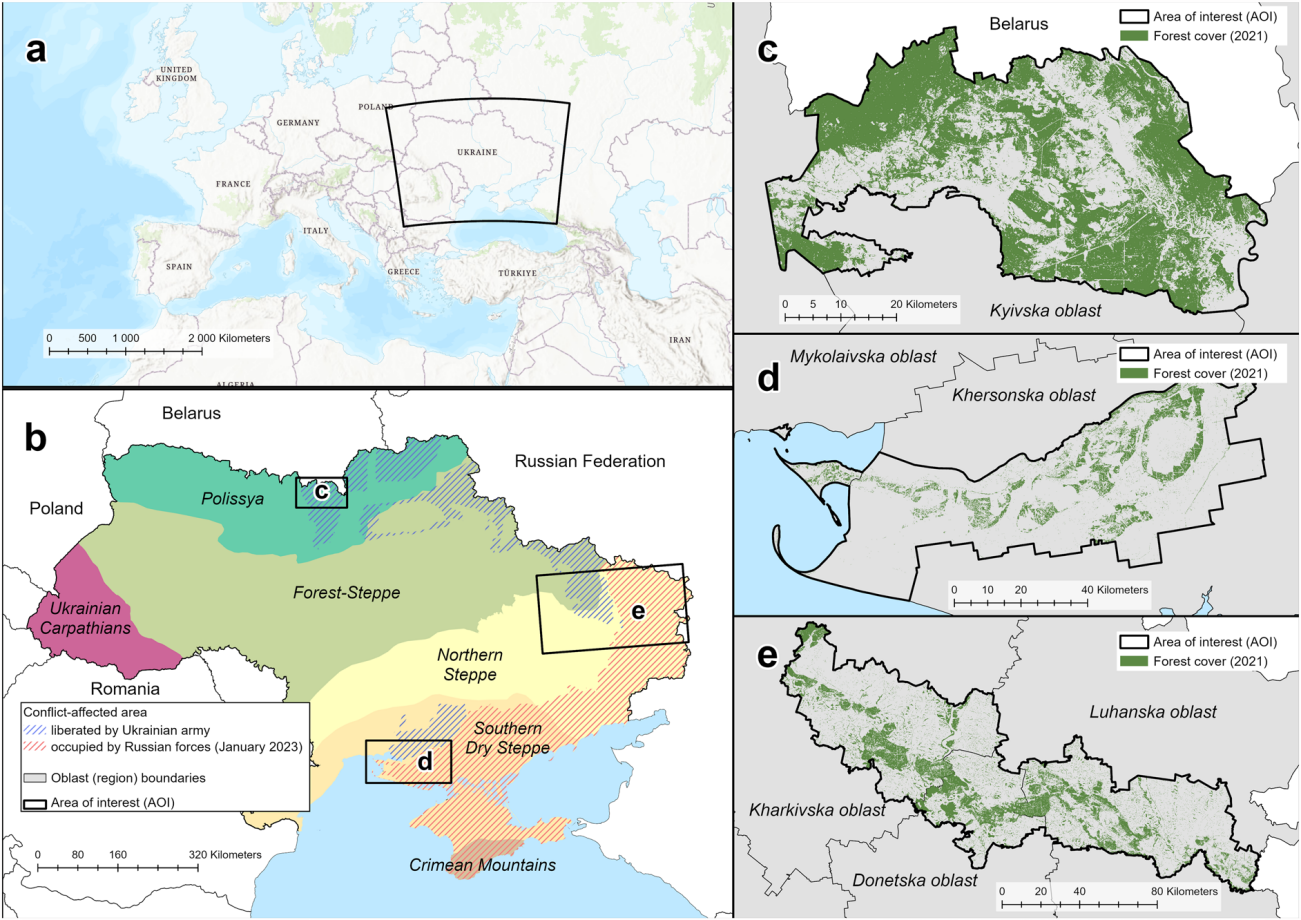
Study location: continental context (**a**); areas of interest (AOI) overlaid on Ukraine’s biogeographical zones (highlighted by different colors) according to Hensiruk^22^ (**b**); CEZ, ‘Kherson’, and ‘East’ AOI ((**c–e**), respectively). The maps are created in ArcGIS Pro 3.2.1.

This study covers three AOIs in Ukraine, Eastern Europe (Fig. 1). In the analysis, we did not include the northern part of Ukraine, except for the CEZ AOI, despite its being under occupation and possessing large forest areas. The northern part of Ukraine was occupied only during late February to early April 2022 and experienced only a few fires in March 2022. From the entirety of northern Ukraine, we selected CEZ as our third AOI as it experienced frequent and occasionally large fires during Russian occupation and the period after its liberation.

All AOIs represent flatland landscapes with elevation not exceeding 300 m a. s. l. ‘East’ AOI encompassed 20 hromadas (counties) in three eastern oblasts (administrative regions) of Ukraine: Kharkivska (eight hromadas, or 5,121 km^2^), Donetska (four hromadas, or 2,110 km^2^), and Luhanska (eight hromadas, or 4,844 km^2^). Its climate is dry, with hot and windy summers, often droughts, and snowless winters. An average annual precipitation is 500 mm, and the average annual temperature is + 10 °C^13^. The region features a fragmented mosaic of croplands and natural or planted forest patches^5^. Scots pine (*Pinus sylvestris* L.) plantations on sandy soils and deciduous forests along the river valleys (basins of Siverskyi Donets and Aidar Rivers) form the forest cover.

The ‘Kherson’ AOI represented seven hromadas in Khersonska oblast and one hromada in Mykolaivska oblast (southern Ukraine), or 4480 km^2^. The local climate is dry, often with droughts that negatively impact crop production in the region. The mean annual precipitation is 408 mm, and the average annual temperature is + 12 °C^23^. To prevent desertification of Khersonska oblast (with vast sandy area locally known as the Oleshky Desert), Scots pine forests were planted before and after the Second World War. Some natural broadleaved forests remain on Kinburn Peninsula in the west of ‘Kherson’ AOI.

The CEZ AOI encompasses the area in the northern part of Kyivska oblast, along the state border with Belarus (2600 km^2^). The mean annual precipitation is 619 mm, the average annual temperature is + 8 °C. Since the Chornobyl nuclear disaster (1986), the forest cover has increased to 59% of CEZ area through natural afforestation of abandoned croplands^24^. The forests in CEZ are mostly Scots pine plantations^25^. Scots pine also naturally replaces former croplands together with silver birch (*Betula pendula* Roth.).

Vegetation cover of all three AOIs is prone to fires. Forests in ‘East’ AOI exhibited megafires in 1996 (region of Kreminna), 2014 (Stanytsia-Luhanska), and 2020 (near Sievierodonetsk). Forests in ‘Kherson’ AOI were severely damaged by fire in the late 2000s and early 2010s. CEZ AOI experienced severe forest fires in 1992, 2015, and 2020 respectively.

Russian military aggression impacted forests of three of the AOIs. CEZ AOI was occupied during late February—early April 2022. Several fires occurred in CEZ in March and May 2022, when firefighting was severely limited by landmine contamination and proximity of the area to the Ukrainian border with Belarus. ‘East’ AOI had already been touched by the war before full-scale 2022 invasion (sharing a contact line with non-government-controlled area in 2014–2022^7^). ‘East’ AOI forests were a battlefield all 2022 year, with multiple fires following shelling. Plantations east to town Kreminna remained (as of September 2023) under Russian control. Forests in the ‘Kherson’ AOI are under Russian occupation since March 2022 (as of September 2023) and have been ravaged by multiple fires following shelling until the late October 2022. Fire suppression efforts in the area were minimal due to its remoteness.

### Land cover classification

**Figure 2:**
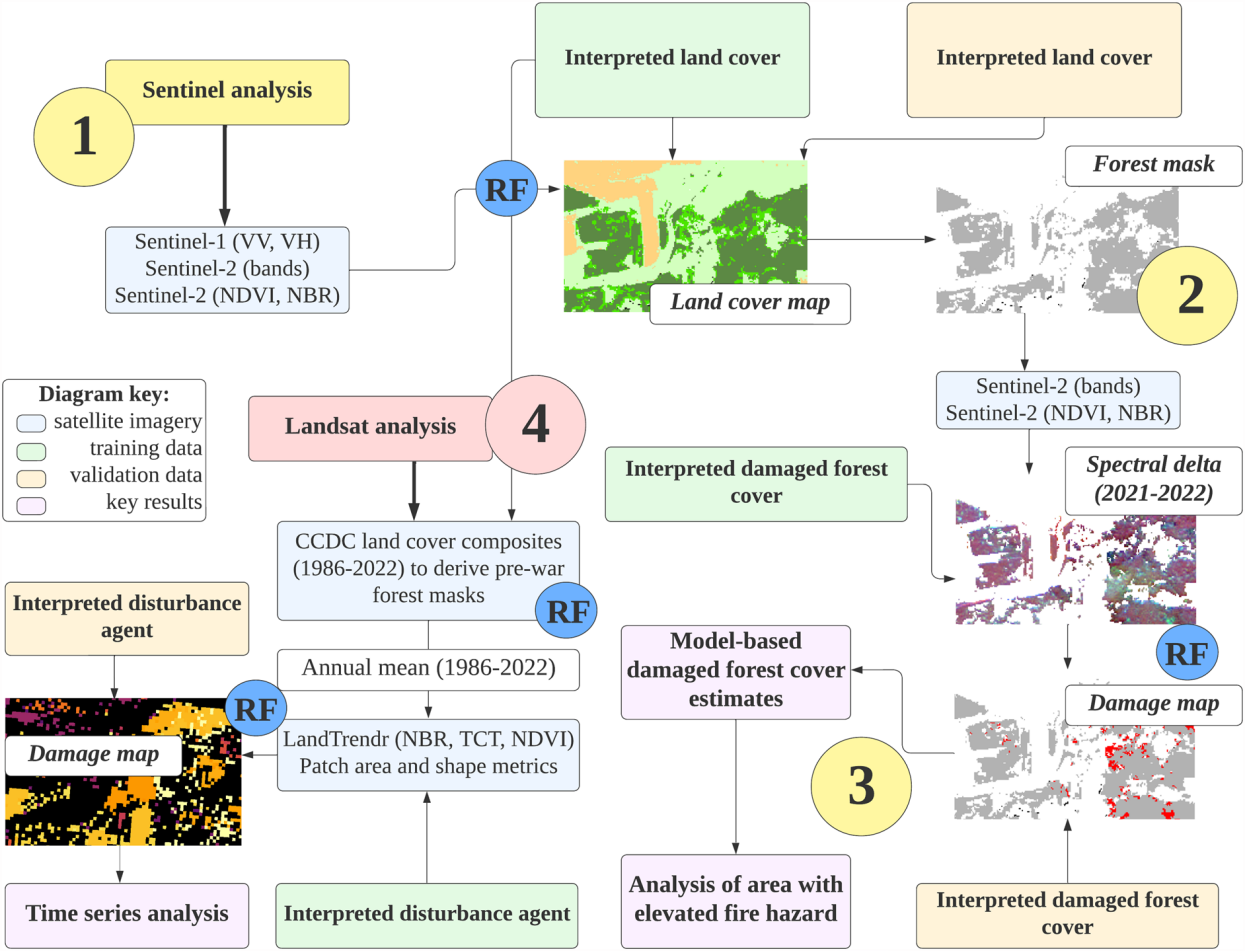
A conceptual flowchart of the study. Step 1 depicts land cover modeling using Sentinel data, step 2 reflects the processes involved in developing damaged forest cover mask. The analysis of elevated fire hazard was carried out in step 3, while step 4 is related to the analysis of pre-war Landsat and climate data.

We used openly available time series of European Space Agency’s Sentinel mission data (Sentinel-1 microwave and Sentinel-2 optical data, resampled to 10 m resolution) to reconstruct 2021 land cover for all three regions. We provide the workflow flowchart in Fig. 2 (with more detailed version in Supplementary Material, Fig. S1). We extracted median composites of all available cloudless images during leaf-on season (Supplementary Material, Table S1), using Google Earth Engine (GEE)^26^. We calculated Normalized Burn Ratio (NBR^27^), Normalized Difference Vegetation Index (NDVI), and a difference between maximum and minimum values during leaf-on season of four Sentinel-2 predictors, i.e. Red, Green, Blue, and Near-Infrared bands. In total, we used 18 predictors (medians of backscatter at two Sentinel-1 polarizations, 10 Sentinel-2 bands, and two vegetation indices; range of four Sentinel-2 band values) to reconstruct 2021 land cover.

For each AOI, we parameterized a Random Forest model (RF^28^) to predict nine land cover classes in each region: bare soil, built-up, burned (before 2022) forest, cropland, forest cover, grassland, water body, wetland, and woodland. To accomplish this task, we manually interpreted land cover in high-resolution imagery (in Google Earth environment) for 814 random locations in ‘Kherson’ AOI, 1,152 locations in the CEZ AOI, and 2,134 locations in the ‘East’ AOI (Supplementary material, Table S1). We used another set of random locations (1981 for the ‘East’ AOI, 831 for the ‘Kherson’ AOI, and 822 for the CEZ AOI) to validate the accuracy of the classification models.

### Estimates of damaged forest cover, based on Sentinel-2 data

We used a set of Word-View (40 cm) true-color scenes available as background imagery in ArcGIS Pro software for the ‘East’ AOI (acquired in August 2022), and multiple Planet (3 m) false-color scenes acquired for all three AOIs in 2022 to calibrate damaged forest cover model. We manually delineated polygons with either totally burned or decolorized tree canopy cover due to fires. In total, we used 1413 polygons: 913 to build the classification model of damaged forest cover and 500 to validate its accuracy of damaged forest cover, and the same number of undisturbed forest polygons.

We used a delta (a net difference) between median composites of Sentinel-2 imagery acquired between 2021 and 2022 (before and during the Russian invasion). To construct these composites, we used median values for all cloudless scenes acquired between 1 August and 30 September for the ‘Kherson’ and CEZ AOIs and 1 July and 30 September for the ‘East’ AOI. We used the RF model to classify disturbances within forested areas that occurred in 2022. Forests harvested prior to February 2022 were excluded from the analyses. We applied filtering to remove single noise pixels from the maps.

We evaluated the accuracy of damage estimates following the established protocol^29^. Specifically, we combined the uncertainty of forest cover prediction for each region with that of RF model predicting damaged forest cover^12^. Finally, we summarized damaged forest cover estimates using 3-km^2^ regular hexagonal grids.

### Analysis of future fire hazard

**Figure 3:**
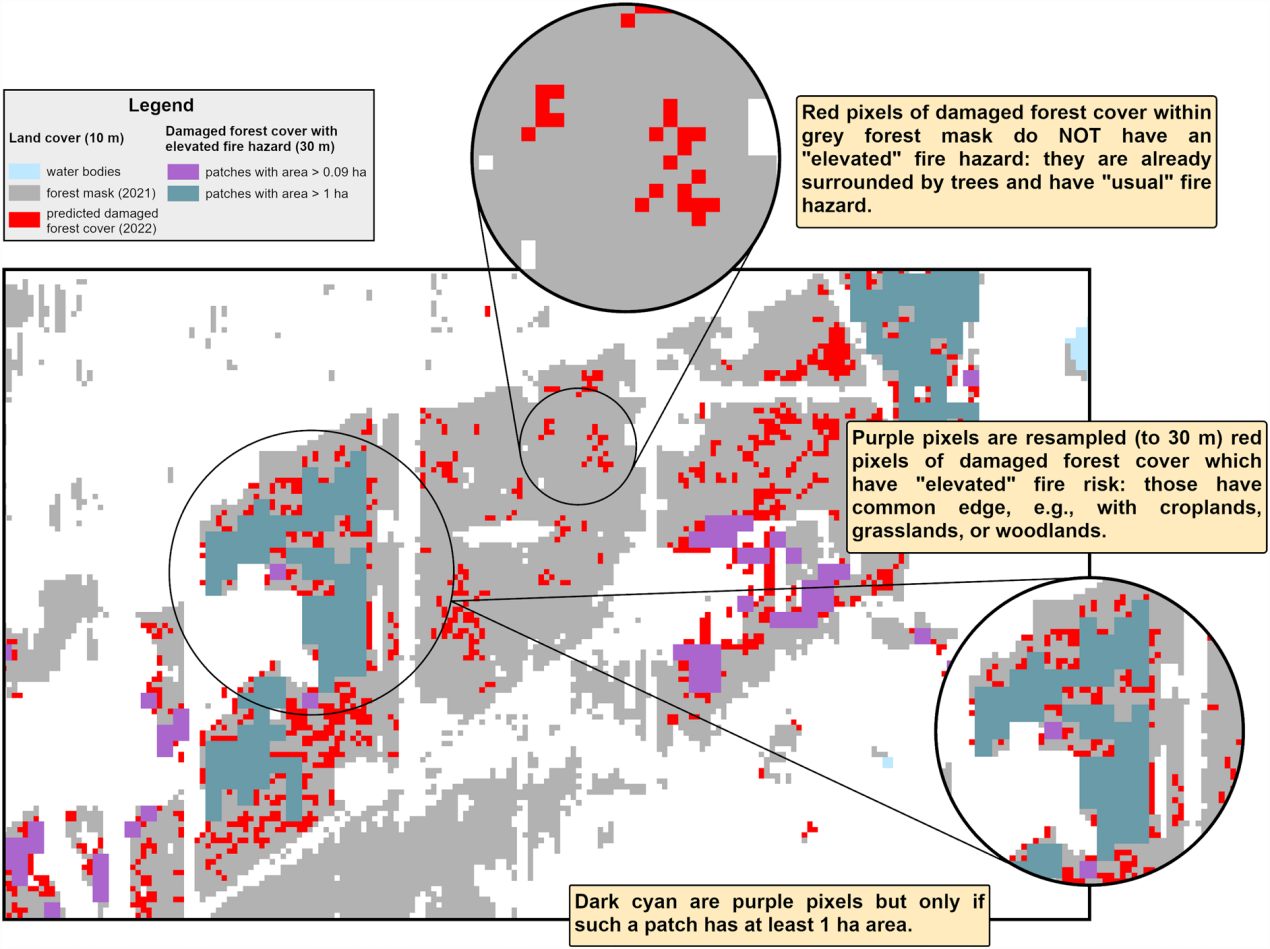
An illustration of ‘elevated fire hazard’ concept used in this study.

To identify forested and damaged areas interfacing with open landscape, we used a damaged forest cover mask based on Sentinel data and resampled it to 30 m resolution. It roughly corresponds (0.09 ha) to the minimum area of a managed forest stand in Ukraine (0.1 ha^30^). We reclassified cover classes into two groups: fire-supporting and fire-inhibiting classes. Cropland, grassland, woodland, and forests burned prior to 2022 were classified as fire-supporting (Fig. 3). Bare soil, built-up, water bodies, and wetlands were classified as fire-inhibiting. Undamaged forest cover was included in the latter. We considered only patches of fire-supporting class with an area of at least one ha. We calculated a Euclidean distance between the edge of the damaged forest patch and the nearest patch of fire-supporting class. Thus, we calculated the area of damaged forest, which is adjacent to a patch of the fire-supporting class. These areas were regarded as forests with ‘elevated fire hazard’. We calculated the total area of such forests in two ways: considering patches larger than 0.09 ha and those larger than 1 ha.

### Analysis of pre-war (with year 2022) forest disturbance regimes

First, we calibrated three AOIs-specific RF models in the GEE environment to map pre-war (before 2022) land cover. We used interpreted land cover data sets (see Supplementary Material, Table S1) similar to those applied to fit Sentinel-based land cover for 2021. Inputs for these RF models were Landsat-based spectral predictors derived by Continuous Change Detection and Classification (CCDC^31^) algorithm. We relied on Tasseled Cap Transformation (TCT, brightness, greenness, wetness) data. We predicted the past land cover for any point of time from 1986 to 2022. Model accuracy was derived by a leave-one-out approach.

Second, we used past forest cover masks provided by CCDC algorithm to remove pre-war disturbances occurred outside the dense tree cover. We used CCDC forest cover masks with 5-year time step (1986, 1990, 1995, 2000, 2005, 2010, 2015, and 2020). We derived inter-annual pre-war (with year 2022) forest disturbance maps using the LandTrendr algorithm^32,33^. We mapped primary (the largest spectral loss segment in the time series for the specific pixel of Landsat imagery) and secondary (the second largest spectral loss segment) forest disturbances. We used the NBR time series to define spectral loss. We selected minimum 0.1 of NBR delta to indicate spectral loss and limited the duration of spectral loss segment by 1–3 years. Single pixels classified as disturbed were considered as the noise and removed. We extracted several raster layers derived by LandTrendr algorithm to classify forest disturbances on causal agents. Those are magnitude (delta) of NBR, NDVI, and TCT bands; NBR value prior to disturbance; duration of segment (1–3 years); disturbance rate (NBR magnitude divided by duration); patch area (area of pixel patch sharing the same year of disturbance); patch shape (value of fractal index of pixel patch sharing the same year of disturbance). The last two metrics were calculated using R package *landscapemetrics*^34^.

We reconstructed three causal agents of pre-war (with year 2022) forest disturbances: stand-replacing fire (SRF), stand-replacing harvest (SRH), and non-stand-replacing (NSR). NSR included low-severity wildfires and fires after shelling (with NBR magnitude < 0.25), pest outbreaks, diseases, drought, wind, and other abiotically induced tree mortality. We collected 1,717 SRF polygons in all three AOIs, 606 SRH polygons in the ‘East’ and CEZ AOIs, and 1,603 NSR (150 polygons representing bark beetle outbreaks in the CEZ AOI, and 1,453 polygons representing low-severity fires after shelling in the ‘East’ and ‘Kherson’ AOIs) polygons. We used information from local forest managers, Google Earth platform, pre-war Landsat, Sentinel-2, and, more recently, Planet and World-View images to delineate these polygons and attribute the causal agents. For validation, we set aside a random subset of 20% polygons of each category, with the rest of the data being used to calibrate the RF classification model.

We calculated the area of disturbed forest cover by causal agents and AOIs for the period 1986–2022. To test for differences in disturbance rates among AOIs (H1), we calculated *z*-score for each year using disturbed area estimates and defined 3 as threshold for abnormal years in causal agent- and AOI-specific time series.

To relate the amount of burned areas to the pre-war fire activity, we regressed proxies of fire weather against the record of burned areas (SRF-attributed disturbances) for each AOI over 1987–2021. We predicted that area for the year 2022 to compare with mapped SRF-attributed area. We collected fire weather data from five points regularly distributed within each of the three AOIs. We used Global Fire Weather Database (GFWED) as a source of daily fire weather data (GFWED^35^). As the exact dates of fires were not known, we aggregated the weather data at the seasonal scale, calculating the average values of Initial Spread Index (ISI) and Drought Code (DC) over the period March through September (inclusive). The period broadly corresponds to the fire season in the areas studied. In addition, we calculated *z*-score for each year using AOI-specific Fire Weather Index (FWI), ISI, and DC time series, and defined 2 as threshold for abnormal years (given that there were no years with *z*-scores ≥ 3).

The data, code and resulting raster layers are available at Zenodo repository (https://zenodo.org/record/8132956). The maps are published at GEE platform as a web-application.

## Results

### Damaged forest cover estimates

**Figure 4:**
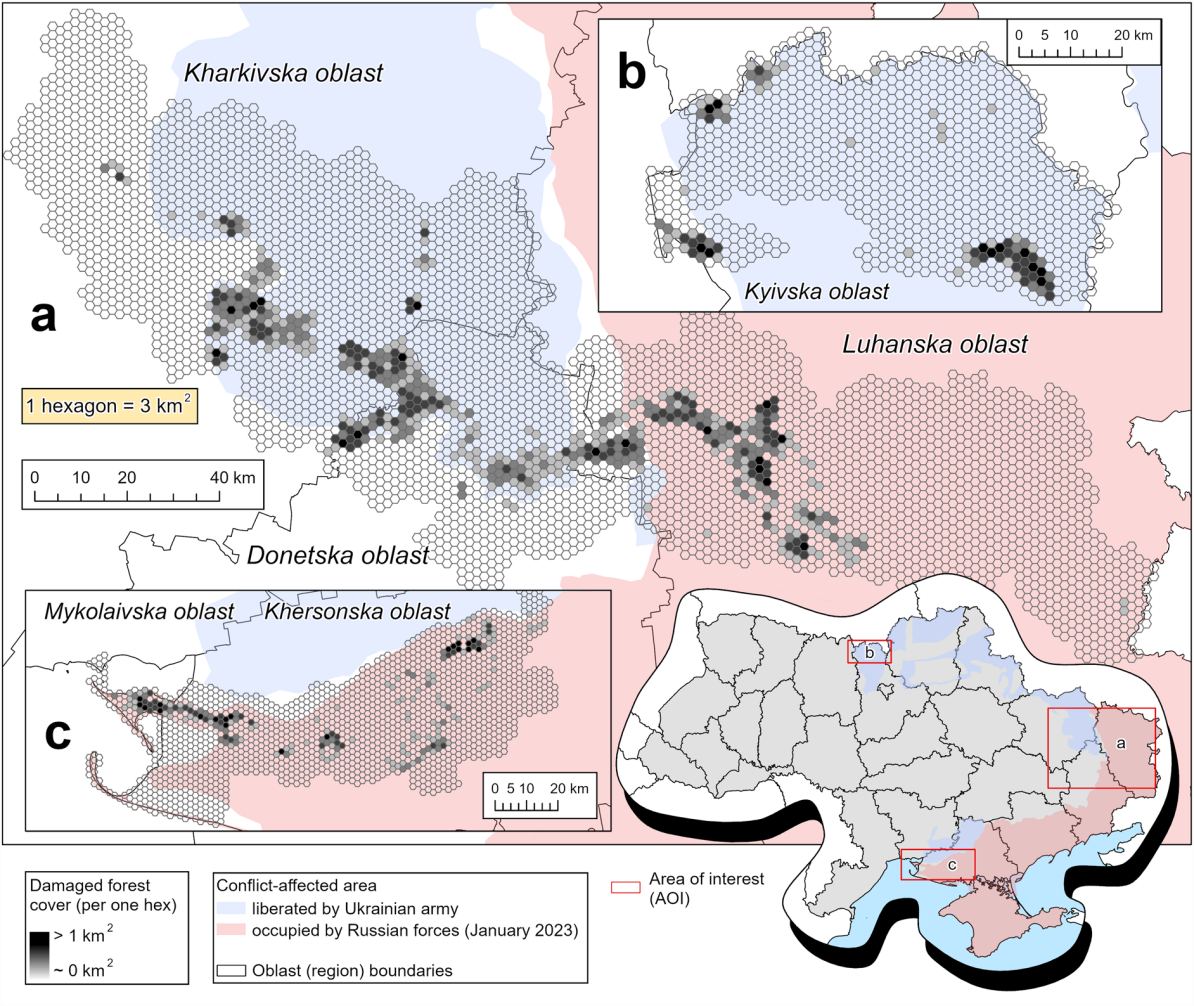
Hexagonal grids of damaged forest cover estimates. There are the following AOIs on the map: ‘East’ (**a**), CEZ (**b**), and ‘Kherson’ (**c**). The maps are created in ArcGIS Pro 3.2.1.

Total damaged forest cover area in the ‘East’ AOI for the period April-September 2022 was 24,180 ± 4715 ha (9.3% ± 1.8% of forest cover in the ‘East’ AOI). Most of detected damage was concentrated in forests around towns Izium and Balakliia (Kharkivska oblast, Fig. 4), near the town Lyman (Donetska oblast), and around the settlements of Kreminna, Rubizhne and Sievierodonetsk (Luhanska oblast). These forests were under active Russian offensive with shelling during the whole study period.

The model estimate for the ‘Kherson’ AOI is 7293 ± 1925 ha (15.7 ± 4.1% of local forest cover). Forests in Kinburn Peninsula (western part of Khersonska oblast) were ravaged the most: fires following shelling could not be efficiently suppressed due to the remoteness of the area. Forest patches along the Oleshky Desert were damaged mainly by low-severity fires.

The damaged forest cover area in the CEZ AOI was estimated as 7116 ± 1274 ha (5.0% ± 0.9% of local forest cover). The large wildfire that happened in May 2022 (southeast of the CEZ AOI) contributed the most to this estimate^36^. Southwestern part of the CEZ AOI was burned in March 2022 while being under Russian occupation, and smaller burned sites were detected in the central (occupied at that time) and western (liberated) parts of the CEZ AOI.

The land cover models parameterized on pre-war data (2021) showed good overall accuracies (for the ‘East’ AOI—81.6%, ‘Kherson’ AOI—75.7%, CEZ AOI—80.7%). The user’s accuracies of forest mask were 79.2% for the ‘East’ AOI, 78.7% for the ‘Kherson’ AOI, 92.0% for the CEZ AOI. Sentinel-1 backscatter predictors were the most important in RF land cover models (Supplementary Material, Figs. S2–S4). Our binary damaged forest cover RF model showed 95.8% overall accuracy (details on validation of this and other RF models are provided in Supplementary Material, Table S2). Infrared predictors contributed the most to the model fit (Supplementary Material, Fig. S5).

### Elevated fire hazard in damaged forests

The ‘Kherson’ AOI featured the highest proportion of damaged forest cover with elevated fire hazard: 89% of damaged patches having an area above 0.09 ha. In damaged forests of AOI ‘East’ and the CEZ AOI, 70% of the forested area was under elevated fire hazard. If considering only patches of damaged forest cover with area 1 ha or larger, these estimates were lower: 75% for the ‘Kherson’ AOI, 59% for the CEZ AOI, and 67% for the ‘East’ AOI.

### Analysis of pre-war (with year 2022) forest disturbances

**Figure 5:**
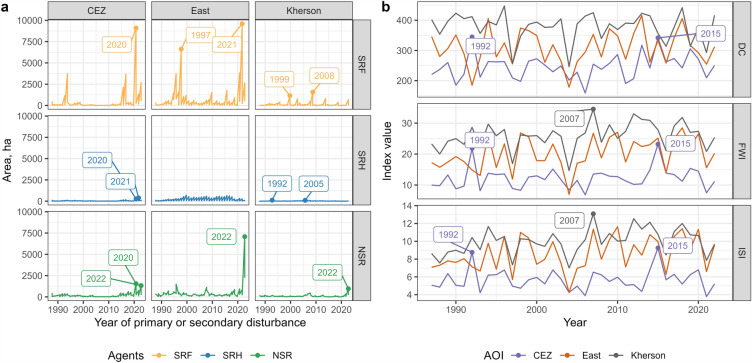
Time series of pre-war and war-related (since 2022) forest disturbances by AOIs and causal agents (**a**) and AOI-specific time series (**b**) of climatic indices (*FWI* fire weather index, *ISI* initial spread index, *DC* drought code). Labeled dots indicate the years where AOI- and agent-specific disturbed area or mean value of climatic index shows significant excess.

During 1986–2022 period, primary and secondary forest disturbances were detected in 97,191 ha in the ‘East’ AOI (37.6% of 2021 forest cover), in 46,012 ha in the CEZ AOI (32.2% of 2021 forest cover), and in 18,494 ha in the ‘Kherson’ AOI (39.8% of 2021 forest cover, Fig. 5). Models based on LandTrendr data clearly captured megafires occurred in 1992 (the CEZ AOI), 1997 (the ‘East’ AOI), 2015 (the CEZ AOI), 2020 for both the CEZ and ‘East’ AOIs) that happened during the study period, except the Kherson fires of 2006. A possible explanation can be that these disturbances occurred mostly in non-dense woody cover (shrubland, young tree stands) while the maps are filtered by pre-war masks of dense forest cover. The protocol based on LandTrendr reported disturbances from late August of the current year as occurring in the following year. For instance, this situation can be observed for SRF in 2021 for the ‘East’ AOI (2020 megafire happened in late September), similar situations for 1997 Kreminna fire in the ‘East’ AOI and 2007 fires over the ‘Kherson’ AOI.

Our past land cover models based on Landsat data and CCDC algorithm produced forest masks with good balanced accuracies (87.3% for the ‘East’ AOI, 86.2% for the CEZ AOI, 84.5% for the ‘Kherson’ AOI). LandTrendr-based RF model for disturbance causal agents’ classification showed 95.9% overall accuracy on the validation data set. An NBR value prior to disturbance was the most important predictor (see Supplementary Material, Fig. S6). Tasseled Cap Brightness and patch shape index were highly instrumental to distinguish SRH from other causal agents. The patch area improved the correct classification for SRF agent, and segment duration supported the more accurate classification of NSR disturbances.

According to the analysis based on *z*-score, the year 2022 was abnormal for all three AOIs in terms of NSR disturbances (Fig. 5a). We did not reveal any excess also for SRF disturbances, which does not support our hypothesis H1. While most of the shelling-mediated (having rather small patch size and visually scattered spatial pattern) fires in ‘East’ and ‘Kherson’ AOIs were attributed to NSR causal agent, no ‘large’ fires (SRF) occurred on significantly larger areas exactly in 2022. No fire weather anomalies were detected for the year 2022 according to FWI, ISI, and DC time series (Fig. 5b). A few specific years with abnormal drought (e.g., 2007 in the ‘Kherson’ AOI or 1992 and 2015 in the CEZ AOI) correspond to severe wildfire events.

Predicted values of the burned areas (SRF regressed from ISI and DC time series) were below the values observed in 2022 in all three AOIs. Compared to SRF-attributed disturbances in the ‘East’ AOI (3,187 ha in 2022), only 1,319 ha were predicted by DC metric (1,318 ha). A similar pattern was observed for the ‘Kherson’ AOI (299 ha by DC-based prediction, 241 ha by ISI compared to 732 ha of mapped SRF disturbances) and the CEZ AOI (906 ha by DC, 766 ha by ISI compared to 3,211 ha mapped by LandTrendr). We noted, however, that fire weather proxies were poor predictors of the area burned in all three AOIs.

## Discussion

### Estimates of damaged forest cover in 2022

We provide the first spatially explicit estimate of damaged forest cover in Ukraine for April-September 2022. In total, three AOIs accounted for 38,589 ± 7914 ha of forest cover damaged during the study period. A vector map of burned protected areas in Khersonska oblast (https://arcg.is/18v0ji0), the one of a few publicly available datasets on forest disturbances in the studied areas, broadly supports our assessment. Specifically, it is in line with our estimates for that AOI, which support our hypothesis (H2) regarding higher relative damage to the ‘Kherson’ AOI forests (15.7 ± 4.1% of pre-war forest cover) compared to the ‘East’ AOI (9.3 ± 1.8%) or the CEZ AOI (5.0 ± 0.9%). The estimate of CEZ area burned in 2022^36^ is reported as three times higher than our estimate (22,171 ha vs. 7116 ha). However, visual inspection of the map published in that report reveals that most fires occurred in CEZ were grassland fires.

Our findings are in disagreement with statements by Ukrainian officials reporting that around 3,000,000 ha of forests in Ukraine are “impacted” (i.e., damaged or contaminated by UXO^37^). This estimate has been likely based on a simple approach of overlaying layers of forest cover in occupied and recently liberated areas. Due to this, it did not represent the area directly affected by shelling or active battles nor did it estimate explicitly the area of landmine fields.

While the current (as for September 2023) frontline stretches beyond ‘Kherson’ and ‘East’ AOIs, it is the forests of these two AOIs that most of the damage occurred^4^. Damage to shelterbelts and urban forests scattered along the frontline (and outside our AOIs) could also be substantial. For example, multiple fires in forest patches were detected on satellite imagery in Autumn 2022 around the Zaporizka nuclear power plant, Enerhodar City. The spatial analysis of such vegetation patches could be challenging due to their sparseness and high level of fragmentation.

### Pre-war (with year 2022) forest disturbances

Shelling-caused ignition was a major factor driving NSR disturbances at large (up to 7000 ha in ‘East’ AOI) areas in the war-affected AOIs, being cause of ignitions inside the forest stands. We expected a higher excess of disturbed area in 2022 for the ‘Kherson’ AOI (hypothesis H2) compared to other AOIs but did not observe any significant differences between this and other AOIs. War-related disturbances were detected at abnormally high rates in each of studied AOIs. The ‘East’ AOI was a battlefield during the whole 2022, and local forests are under constant ravaging as for September 2023 as it can be monitored on recent Sentinel-2 and Planet images. The area of NSR disturbances there and in Kherson’s AOI were equally high. Low intensity fires were also detected across the CEZ AOI in 2022, being also an unusual phenomenon for a local pre-war disturbance regime.

Shelling was also the cause of various forms of direct (‘mechanical’) damage to forests in the ‘East’ AOI. Our LandTrendr-based model classified several large, disturbed patches as SRF in some broadleaved (*Quercus* spp.-dominated) forests in Donetska oblast of the ‘East’ AOI. However, damage to forest cover was not visible in these forests on satellite images from 2023. Consultation with volunteers and members of the Ukrainian Armed Forces who were present in these areas during the spring–summer of 2022 indicated that these forests likely experienced temporary defoliation due to extremely intense outgoing artillery barrages from adjacent Russian positions. If this is indeed the case, we would expect the majority of these stands to recover. It is still uncertain what level of delayed mortality will be observed in the years following these NSR disturbances.

### Utility of remote sensing data

The satellite-based approach to map forest disturbances (e.g., fires or tree harvest) using open Sentinel data is of high value in Ukrainian conditions. However, cloud, noise, and unrelated disturbances (such as clear-cuts before Russian invasion, but after 2021 leaf-on season) should be filtered, and large-scale damaged forest cover mapping thus must rely on well-established semi-automatic algorithms. Importantly, additional tree species classification (omitted in this study) should be conducted: our elevated fire hazard approach is designed for coniferous forests, while still some deciduous stands (e.g., along Siverskyi Donets River) could be damaged as well.

**Figure 6:**
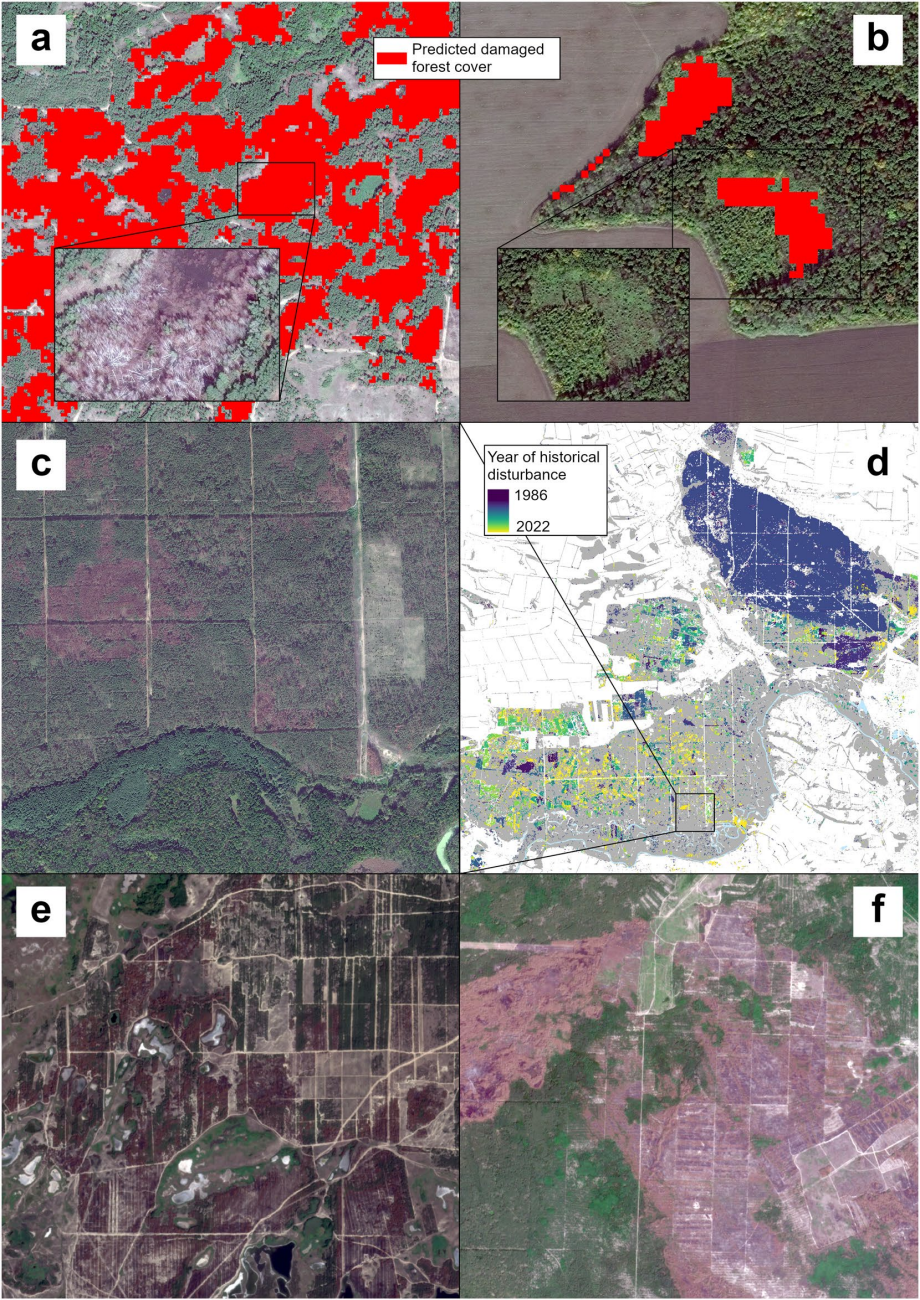
Detected severely burned forests west to town Izium, the ‘East’ AOI (**a**); tree harvest detected by Sentinel-based model near Sievierodonetsk City, the ‘East’ AOI (**b**); damaged Scots pine forests south-east to Kreminna, the ‘East’ AOI (**c**); larger look at Kreminna area with LandTrendr-derived pre-war disturbance map, with forest landscape totally burned in 2007 (**d**); complex mosaic of live, burned forests, and grassy vegetation in Kinburn Peninsula, the ‘Kherson’ AOI (**e**); destroyed dense Scots pine monocultures in CEZ AOI after May 2022 wildfire in a place devastated by megafire in 1992 (**f**). Image sources: World-View 3 (22 August 2022)—(**a–c**) planet (09 September 2022), (**e**) planet (06 June 2022), (**f**) The maps are created in ArcGIS Pro 3.2.1.

Our binary classification model (damage or no damage in forest cover) achieved good overall accuracy (95.8%). This result was in line with multiple investigations which used Sentinel-2 data to map burned areas (e.g.,^16^), tree harvest (e.g.,^38^), or tree mortality caused by biotic agents (e.g.,^39^). Our model relies on a simple difference in spectral characteristics of the AOIs prior to (2021) and following the invasion (the late phase of the leaf-on season 2022). We successfully mapped burned areas (Fig. 6a) and illegal logging in occupied areas close to the current frontline (Fig. 6b). We assumed that many forests burned with low-severity fires following the shelling and remained undetected by our model. We expect that trees in these patches will mostly survive^13^ and such areas will not be subject to salvage logging. The latter is important as in this study we also focus on damaged forest cover which has elevated fire hazard if harvested and replanted by Scots pine monocultures.

We provided spatially explicit information on primary and secondary (according to LandTrendr approach^32^) forest disturbances. We achieved good accuracy for all three classes of disturbance causal agent. While previous studies solely relied on the NBR magnitude indicator of spectral change caused by disturbance^14,40^, we also extracted fitted data for other predictors. Here, tasseled cap brightness was extremely useful for distinguishing tree harvest (SRH) from other disturbance agents: clear-cut sites become literally ‘lighter’ for satellite sensors, while burned tree canopies range from red (slightly ‘lighter’ than healthy dark-green foliage) to black (far ‘darker’ in visible range of electromagnetic spectrum). Adding non-spectral landscape metrics (patch area and shape fractal index) contributed to higher accuracy of our classification model. Earlier, complex focal landscape configurations were used to delineate harvest disturbances from those caused by wind and pest outbreaks^41^. In our study, the fractal shape index of disturbed patches was mapped with lower values for simple rectangular patches (clear-cuts, or SRH), while naturally occurring SRF and NSR disturbances had irregular shape and thus higher values of this index. The patch area contributed to distinguishing generally larger SRF from small low-severity fires following shelling (attributed to NSR agent).

### Future fire hazard and management implications

We speculate that the fire hazard of studied AOIs will remain high even in recently burned areas. Due to UXO contamination, many burned pine plantations remain un-salvaged^4^. This will result in large accumulations of woody fuel, both standing and on the ground, which will soon be supplemented by herbaceous post-fire vegetation that is highly flammable in dry periods^9^. There is a history of such areas re-burning in the CEZ AOI several years after catastrophic fires^8,12^.

Fires of 2022 increased the level of forest cover fragmentation in all of the AOIs, and particularly so in the ‘Kherson’ AOI. The 89% of the patches in this AOI have elevated fire hazard as they border with grasslands, croplands, and woodlands (Fig. 6e). Due to their UXO contamination, fire suppression in such forests will remain highly challenging. The remaining patches of unburned, dense pine plantations will face high fire hazard due to an increased risk of fire spreading into them from the surrounding vegetation. This consideration laid ground for formal management recommendation to monitor and reduce fuel loads in 500 m wide zones around forests surrounded by croplands, woodlands, and pastures^42^.

Artificial regeneration of burned areas does not guarantee reduction in fire hazard. Newly established Scots pine plantations are highly flammable, and once they reach a height of 6 m, they act as ladder fuels for neighboring mature pine plantations (Fig. 6f). Structurally homogenous stands of high-density pine plantations (even of varying ages) are not an ecologically resilient in water-stressed sandy soils of eastern Ukraine^9^. Crown fires spread through such forests extremely well due to their almost uninterrupted canopies. Survival of individual pines in these dense artificial stands after high-intensity ground fires is often low, apparently due to low bark thickness of these relatively young (< 30 years) trees^43^. Studies have shown that post-fire recovery of these stands may be hampered by high levels of competition for soil moisture with grass vegetation^44^.

In Ukraine, high-density (up to 10,000 tree seedlings per one ha) pine plantations have also experienced major drought-related dieback events, suggesting their sensitivity to environmental variability^10,19^. Regeneration of areas burned during 2022 by Scots pine will make them prone to future forest fires. To address these concerns, studies have recommended transitioning fire-damaged landscapes dominated by pine plantations to a mosaic of pine plantations of varying densities (from typical high-density plantations on moist sites to widely spaced ‘woodland’ structures on the driest sites) and mixed pine-deciduous stands^9^. Other options to manage fire risks could include belts of planted deciduous trees that break up continuous expanses of pine, and open herbaceous communities (‘sandy steppe’) in extremely dry and unproductive sites. In fact, forest fragmentation created by war-related disturbances could be a starting point in developing such mosaic. We speculate that recently fragmented forests could benefit from only partial regeneration of disturbed patches, with some of them being kept as fire breaks with low fuel loads. This should reduce fuel contiguity that facilitate rapid fire spread.

Controlling tree density and forest edges could be important elements of new management policies^45^. In the case of density, we propose gradual thinning to make stand canopies sparser to facilitate development of thicker and better insulating bark. We expect higher survival rate of trees in ground fires and better suppression possibilities during crown fires in thinned stands^43^. It is likely that low density pine stands were common as a part of natural vegetation across all three AOIs prior to the advent of intensive plantation silviculture. Edges of forests bordering agricultural fields and residential areas are typical ignition sources and their management may include control of stand densities and fuel loads, in particular—ladder fuels^6^. Such practices would then be similar to those applied to ‘urban-wildland interface’ in North America^11^.

Local topography may help increase forest resilience against fires. For example, one of the largest pine forested areas in the ‘East’ AOI southwest of the city of Kreminna is bordered to the east and south (the directions from which the predominant winds of that region typically bring fires) by the Siverskyi Donets River and its deciduous forests in the floodplain and on lower terraces (Fig. 6c). This helps to explain why these forests were virtually untouched by forest fires that occurred during 1991–2021, when almost all other pine forests in the ‘East’ AOI were affected by fires^7^. The area of Scots pine plantations of comparable scale to the northeast of Kreminna that had no buffer separating it from adjacent agricultural fields, experienced fire that burned 5,750 ha in 1997 (Fig. 6d).

Our study did not discuss the role of secondary disturbances, e.g. higher rate of windfalls in the forests with increased edge-to-size ration nor the effects of changes in hydrology due to destruction of Nova Kakhovka dam in June 2023. Their impacts will be likely long-term and would require an equally long monitoring program to adequately evaluate them. The war in Ukraine is ongoing and the estimates reported in the study are to be updated as soon as new data become available. At the time of submission of this paper, abundant video material posted by combatants and volunteers from both sides in 2023 indicates wide-spread damage to mature pine forests in the Kreminna area of the ‘East’ AOI due to incoming artillery strikes on Ukrainian positions in the area. This damage resulted in broken crowns and treefalls, and led to high crown mortality, resembling that after strong wind episodes.

We call for the development of a program for long-term monitoring of damaged areas using field inventories (where possible) and remote sensing tools. There is an urgent need for a new conceptual framework to guide regeneration programs that would consider landscape context and future fire risks, particularly for southern and eastern part of Ukraine. Remotely sensed data provide strong support to future forest planning in Ukraine that should account for the landscape-level distribution of fuels, risk of uncontrolled fire spread, and UXO contamination.

## Conclusions

Remote sensing data remains the only feasible information source on the extent of damage to Ukraine’s forests brought about by Russia’s invasion. We found a significantly higher proportion of damaged forest cover area in the ‘Kherson’ AOI (15.7% of forests with 2021 as baseline) compared to ‘East’ AOI (9.3%) and CEZ AOI (5.0%). We link this to shelling-induced fires, as indices of natural fire weather for the year 2022 were moderate and could not predict the scale of burned area within the studied AOIs. Within the ‘Kherson’ AOI, the landscape mosaic of various land uses appears more diverse, and forests are more fragmented: 89% of damaged forest patches will likely have elevated fire hazard (compared to 70% in ‘East’ and CEZ AOIs respectively). Recovery of these damaged forests in the post-war period could be compromised with the current management practices promoting forests not resilient to climate-induced stresses. We call for incorporation of “fire-aware” restoration policies into post-war forest management and long-term monitoring of war-affected Ukrainian forests.

### Supplementary Information

Supplementary Information.

## Data Availability

Data and code for analysis (including metadata) are available at Zenodo (https://zenodo.org/record/8132956). The maps are published at GEE platform as a web-application (https://matsalanubip.users.earthengine.app/view/damage-to-forests-in-ukraine-1986-2022).
