# Supplementary material for: War drives forest fire risks and highlights the need for more ecologically-sound forest management in post-war Ukraine
Source: Sci Rep. 2024 Feb 19;14:4131. doi: 10.1038/s41598-024-54811-5 (PMC10876951; doi:10.1038/s41598-024-54811-5)
Supplement: Supplementary file 1 — Supplementary Information. [file 41598_2024_54811_MOESM1_ESM.zip › Supplementary.docx]

Table S1. The detailed description of data used in this study.

| No. | Type of data | Number of points or polygons | Where used | Description |
| --- | --- | --- | --- | --- |
| *Raster data* | | | | |
| 1 | Sentinel-1 | – | Sentinel analysis | Sentinel-1 backscatter C-band data was extracted at two polarizations, VH (vertical transmission – horizontal receiving) and VV (vertical transmission – vertical receiving). These two predictors represent preprocessed (thermal noise removal, radiometric and terrain calibration) raster layers in ground range detection format, then filtered with Lee spatial speckle filter.  Spatial resolution is 10 m. Median composites for leaf-on season (April – September) were extracted using Google Earth Engine (GEE) platform. We used Sentinel-1 data acquired at both (ascending and descending) orbits. |
| 2 | Sentinel-2 | – | Sentinel analysis | We used median composites of all available cloudless images for leaf-on season (April – September). These data are represented by a surface reflectance of optical bands (red, green, blue, near infrared, four red-edge, two short-wave infrared bands) with wavelengths ranging from 492 to 2202 nm. These data were geometrically and radiometrically calibrated on GEE platform.  In addition, we calculated two common vegetation indices, Normalized Burn Ratio (NBR, normalized difference between near infrared and short-wave infrared bands) and Normalized Difference Vegetation Index (NDVI, normalized difference between near infrared and red bands).  Raster layers of red-edge and short-wave infrared bands (20 m spatial resolution) were resampled to 10 m to align with other Sentinel-2 data. This operation was also carried out while calculating NBR.  In total, 12 optical predictors (10 Sentinel-2 bands and two vegetation indices) were used to predict land cover (as for 2021).  Then, extracted median composites in two-month window (August-September for ‘Kherson’ and CEZ AOIs) or three-month window (July-September for ‘East’ AOI) both for 2021 (before invasion) and 2022 (during invasion). We calculated a net delta (difference) between these composites and used it to build damaged forest cover classification model. We selected this period as a late part of leaf-on season (July was included for ‘East’ AOI due to lacking cloudless scenes for later months). |
| 3 | Landsat | – | Landsat analysis | We used annual mean composites of fitted (by a simple geometrical regression, Kennedy et al. 2010) NBR values to segment time series of Landsat (30 m) resolution data. Based on segments defined by NBR time series, time series of Tasseled Cap Transformation bands (brightness, greenness, and wetness) and NDVI were also segmented. These data were used to build LandTrendr-based classification model of disturbance causal agents.  Intra-annual Landsat composites were processed following Zhu et al. (2014) approach based on CCDC algorithm. |
| *Vector data* | | | | |
| 1 | Land cover (2021) in ‘Kherson’ AOI | 814 points | Sentinel analysis | Random set of 711 locations was used to visually interpret land cover (for 10 x 10 m box) using high-resolution imagery available in Google Earth. Additionally, 103 points were manually distributed in underrepresented land cover classes. |
| 2 | Land cover (2021) in ‘East’ AOI | 2,134 points | Sentinel analysis | Random set of 2,000 locations was used to visually interpret land cover (for 10 x 10 m box) using high-resolution imagery available in Google Earth. Additionally, 134 points were manually distributed in underrepresented land cover classes. |
| 3 | Land cover (2021) in CEZ AOI | 1,152 points | Sentinel analysis | Random set of 1,000 locations was used to visually interpret land cover (for 10 x 10 m box) using high-resolution imagery available in Google Earth. Additionally, 52 points were manually distributed in underrepresented land cover classes, and 100 locations were interpreted in a previous field campaign in CEZ (Matsala et al. 2021). |
| 4 | Land cover (2021) in ‘Kherson’ AOI | 831 points | Sentinel analysis | Randomly distributed points for validation. Each point is minimum 100 m far away from any training points (data set No. 1). |
| 5 | Land cover (2021) in ‘East’ AOI | 1,981 points | Sentinel analysis | Randomly distributed points for validation. Each point is minimum 100 m far away from any training points (data set No. 2). |
| 6 | Land cover (2021) in CEZ AOI | 822 points | Sentinel analysis | Randomly distributed points for validation. Each point is minimum 100 m far away from any training points (data set No. 3). |
| 7 | Unburned perimeters for Sentinel damage model | 1,413 polygons | Sentinel analysis | High-resolution imagery available in Google Earth, ArcGIS Pro (true-color World-View, 40 cm), and false-color Planet (3 m) imagery were used to delineate perimeters free of canopy mortality (as for August-September 2022); 913 polygons were used to calibrate model and 500 for validation.  No visual sign of tree canopy damage (burned canopies or other mortality) was observed for this set of polygons. |
| 8 | Burned perimeters for Sentinel damage model | 1,413 polygons | Sentinel analysis | High-resolution imagery available in Google Earth, ArcGIS Pro (true-color World-View, 40 cm), and false-color Planet (3 m) imagery were used to delineate perimeters with clear canopy mortality (as for August-September 2022); 913 polygons were used to calibrate model and 500 for validation.  These polygons represent forest patches with clearly visible tree canopy damage (burned or harvested). |
| 9 | Land cover in Kherson AOI (2012-2022) for CCDC | 911 points | Landsat analysis | Data set No. 1 was used as a baseline for these data. These locations were provided with a date corresponding to available high-resolution image in Google Earth, and some were reclassified or removed. Reclassification or removal was carried out for locations on a margin of different land cover, and is intended to fit Landsat pixel size (30 m) instead of Sentinel pixel size (10 m). |
| 10 | Land cover in East AOI (2012-2022) for CCDC | 2,047 points | Landsat analysis | Data set No. 2 was used as a baseline for these data. These locations were provided with a date corresponding to available high-resolution image in Google Earth, and some were reclassified or removed. Reclassification or removal was carried out for locations on a margin of different land cover, and is intended to fit Landsat pixel size (30 m) instead of Sentinel pixel size (10 m). |
| 11 | Land cover in CEZ (2012-2022) for CCDC | 1,168 points | Landsat analysis | Data set No. 3 was used as a baseline for these data. These locations were provided with a date corresponding to available high-resolution image in Google Earth, and some were reclassified or removed. Reclassification or removal was carried out for locations on a margin of different land cover, and is intended to fit Landsat pixel size (30 m) instead of Sentinel pixel size (10 m). |
| 7 | Stand replacing fires’ (SRF) polygons | 1,717 polygons | Landsat analysis | Historical wildfires were detected using prior knowledge on large fire events, and LandTrendr disturbance maps (with focus on large patches with irregular shape). Landsat, Sentinel-2 and Planet imagery were used to delineate these SRF perimeters. Additionally, fire patches with NBR loss > 0.25 in forests damaged by shelling (2022) and digitized using high-resolution imagery were included in this data set of SRF polygons.  Historical wildfire polygons were digitized in all AOIs, shelling (2022) patches – only for ‘East’ and ‘Kherson’ AOIs. |
| 9 | Stand replacing harvest (SRH) polygons | 606 polygons | Landsat analysis | Historical clear-cuts were detected using LandTrendr disturbance maps (with focus on patches with area below 1 ha (< 10-11 pixels) and rectangular shape), and double-checked with Google Earth historical imagery. SRH polygons were digitized in CEZ and ‘East’ AOIs. |
| 10 | Non-stand-replacing (NSR) polygons | 1,603 polygons | Landsat analysis | Historical NSR events were digitized in CEZ AOI using prior knowledge: 2016 windstorm damage; 2018 bark beetle outbreak; recent tree mortality due to flooding or diseases. Additionally, fire patches with NBR loss between 0.1 and 0.25 in forests damaged by shelling (2022) were included in this data set of NSR polygons. These low-severity fires are not likely to cause the stand replacement naturally. |

Table S2. Summary of model accuracies.

| Model | Accuracies | Validation approach |
| --- | --- | --- |
| Sentinel (2021) land cover model for ‘East’ AOI | 81.6% (OA), 79.2% (UA forest cover), 89.7% (PA forest cover) | Following Oloffson et al. (2014) protocol and using independent data set |
| Sentinel (2021) land cover model for ‘Kherson’ AOI | 75.7% (OA), 78.7% (UA forest cover), 82.8% (PA forest cover) | Following Oloffson et al. (2014) protocol and using independent data set |
| Sentinel (2021) land cover model for CEZ AOI | 80.7% (OA), 92.0% (UA forest cover), 94.4% (PA forest cover) | Following Oloffson et al. (2014) protocol and using independent data set |
| Sentinel (2022) damaged forest cover binary model | 95.8% (OA), 79.2% (UA damaged cover), 74.2% (PA damaged cover) | Following Oloffson et al. (2014) protocol and using independent data set |
| Landsat CCDC land cover model for ‘East’ AOI | 78.4% (OA), 87.3% (balanced accuracy, forest cover) | Leave-on-out approach |
| Landsat CCDC land cover model for ‘Kherson’ AOI | 75.3% (OA), 84.5% (balanced accuracy, forest cover) | Leave-on-out approach |
| Landsat CCDC land cover model for CEZ AOI | 73.2% (OA), 86.2% (balanced accuracy, forest cover) | Leave-on-out approach |
| Landsat LandTrendr historical forest disturbance causal agent model | 95.9% (OA) | Confusion matrix using independent data set |

Note: OA – overall accuracy, UA – user’s accuracy, PA – producer’s accuracy.


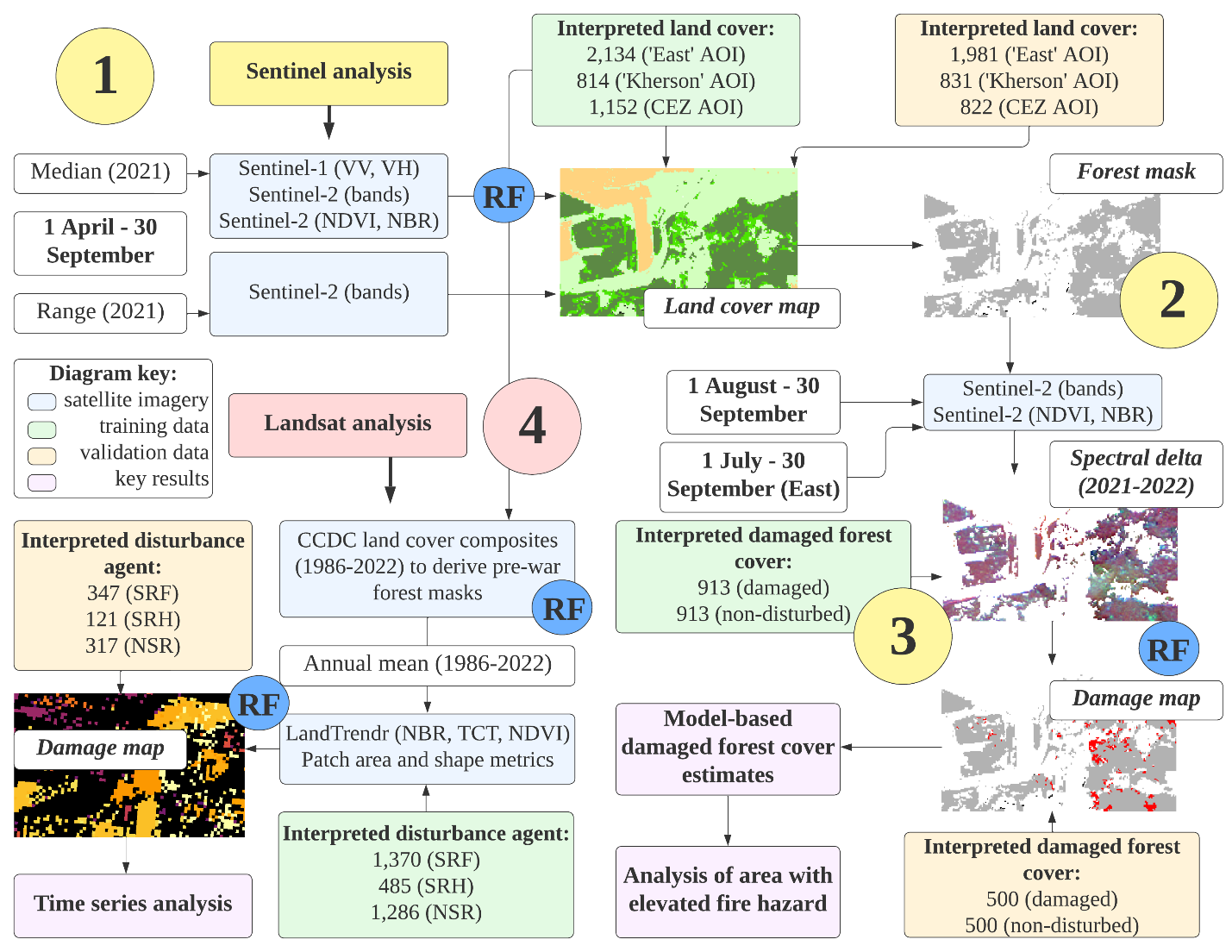


Figure S1. Detailed workflow of the study. Sentinel-based analysis is illustrated at steps (yellow circles) 1-3, and is designed to provide damaged forest cover estimates and analysis of damaged forest cover with elevated fire hazard. Third research question of this study (analyzing pre-war forest disturbance regimes in AOIs) here is linked to step 4 (red circle) and Landsat analysis. A diagram key depicts blocks where satellite data was applied (blue boxes), training (green boxes) and validation (orange boxes) data explanation, and pink boxes reflect key results (three research questions of this study).


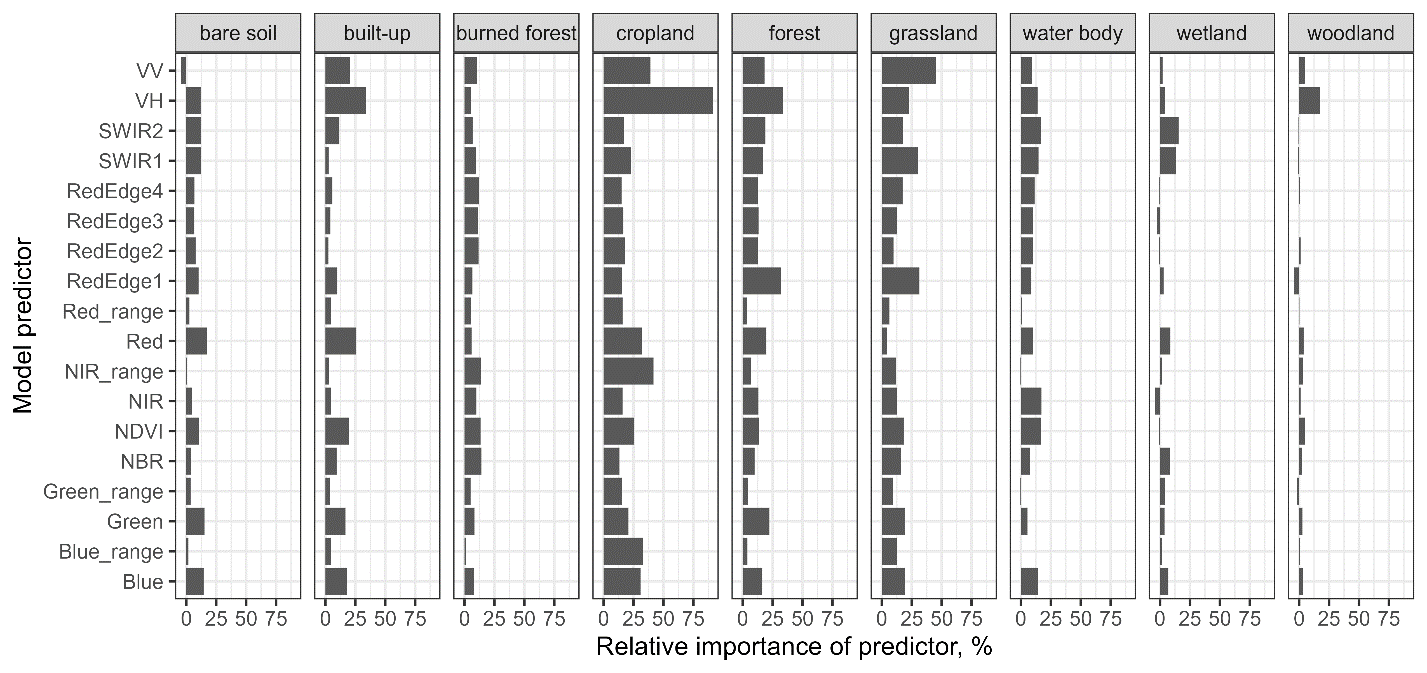


Figure S2. A relative importance of Random Forest (RF) land cover (2021) model for ‘East’ AOI. Model predictor abbreviatures: VV – Sentinel-1 backscatter at vertical-vertical polarization; VH – Sentinel-1 backscatter at vertical-horizontal polarization; SWIR – short-wave infrared; NIR – near-infrared; NDVI – normalized difference vegetation index; NBR – normalized burn ratio.


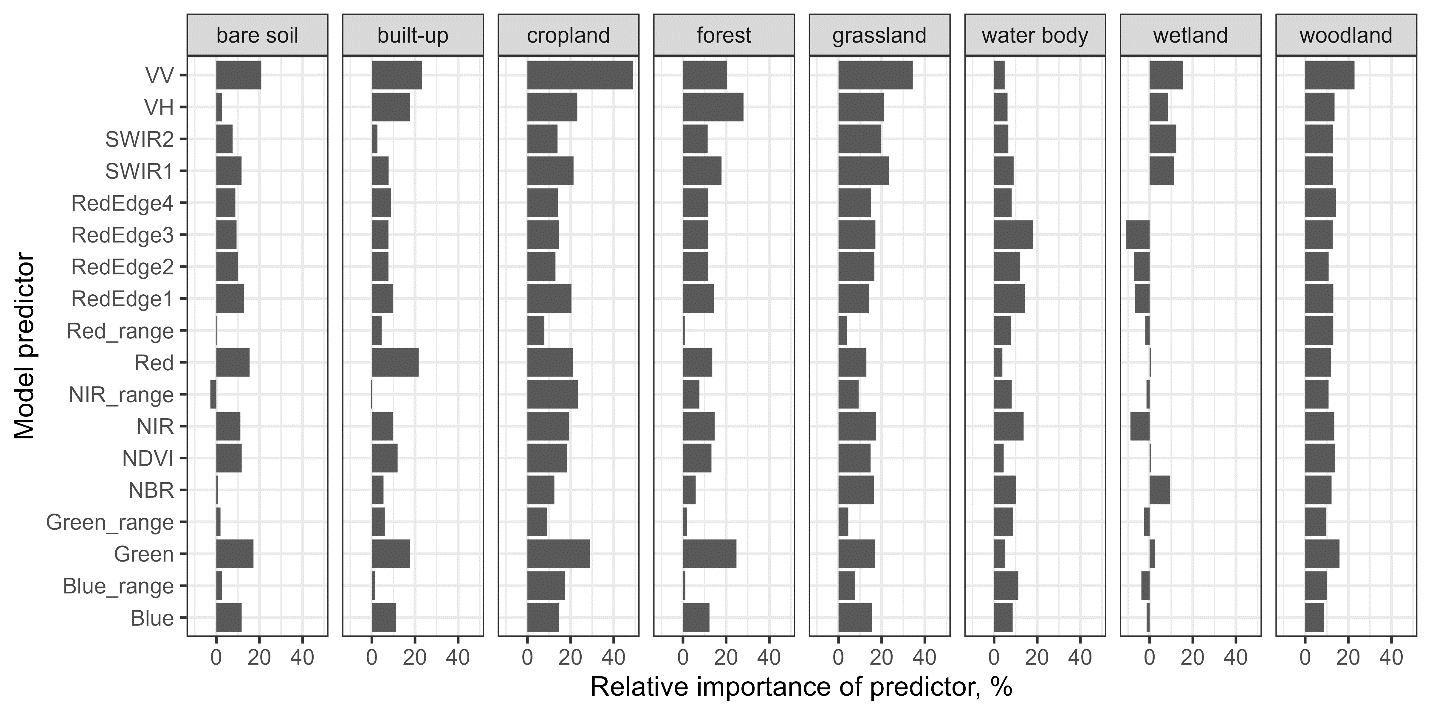


Figure S3. The relative importance of RF land cover (2021) model for ‘Kherson’ AOI. Model predictor abbreviatures are as in Figure S2 caption.


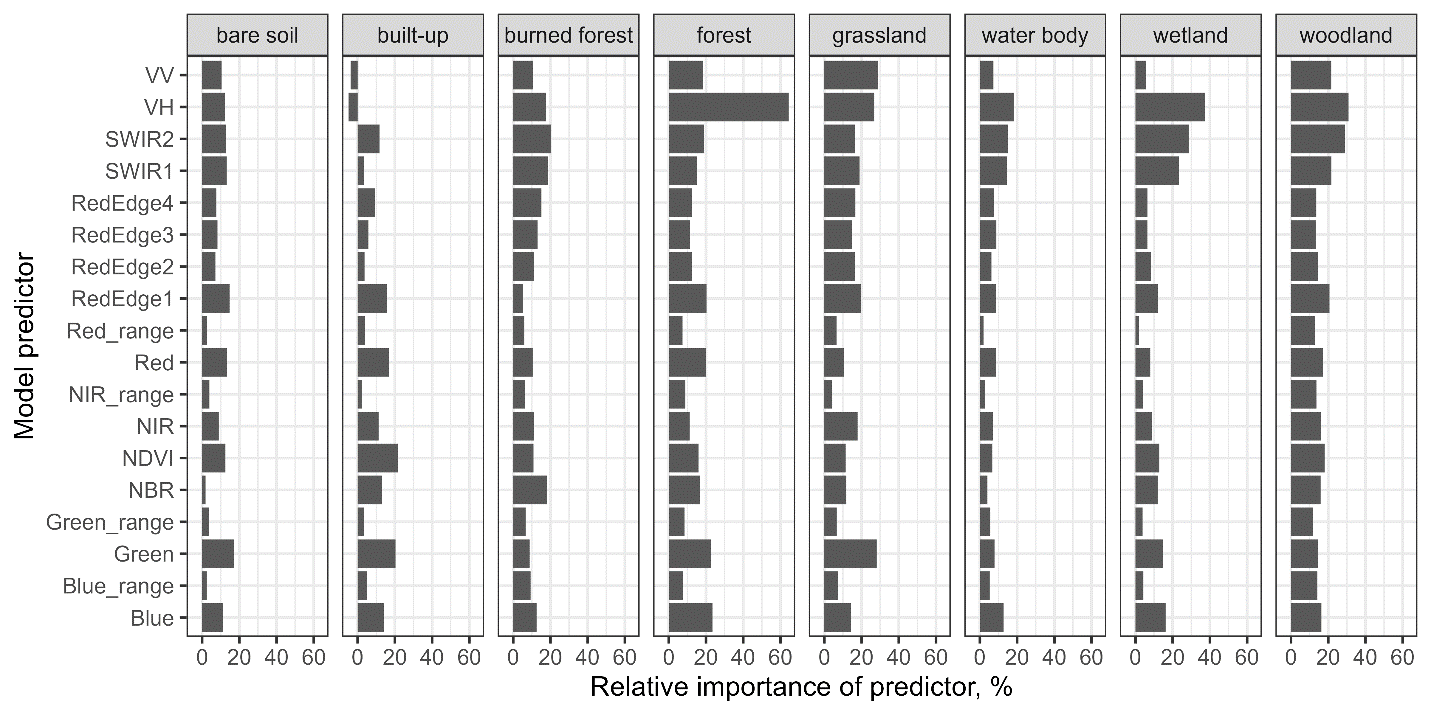


Figure S4. The relative importance of RF land cover (2021) model for CEZ AOI. Model predictor abbreviatures are as in Figure S2 caption.


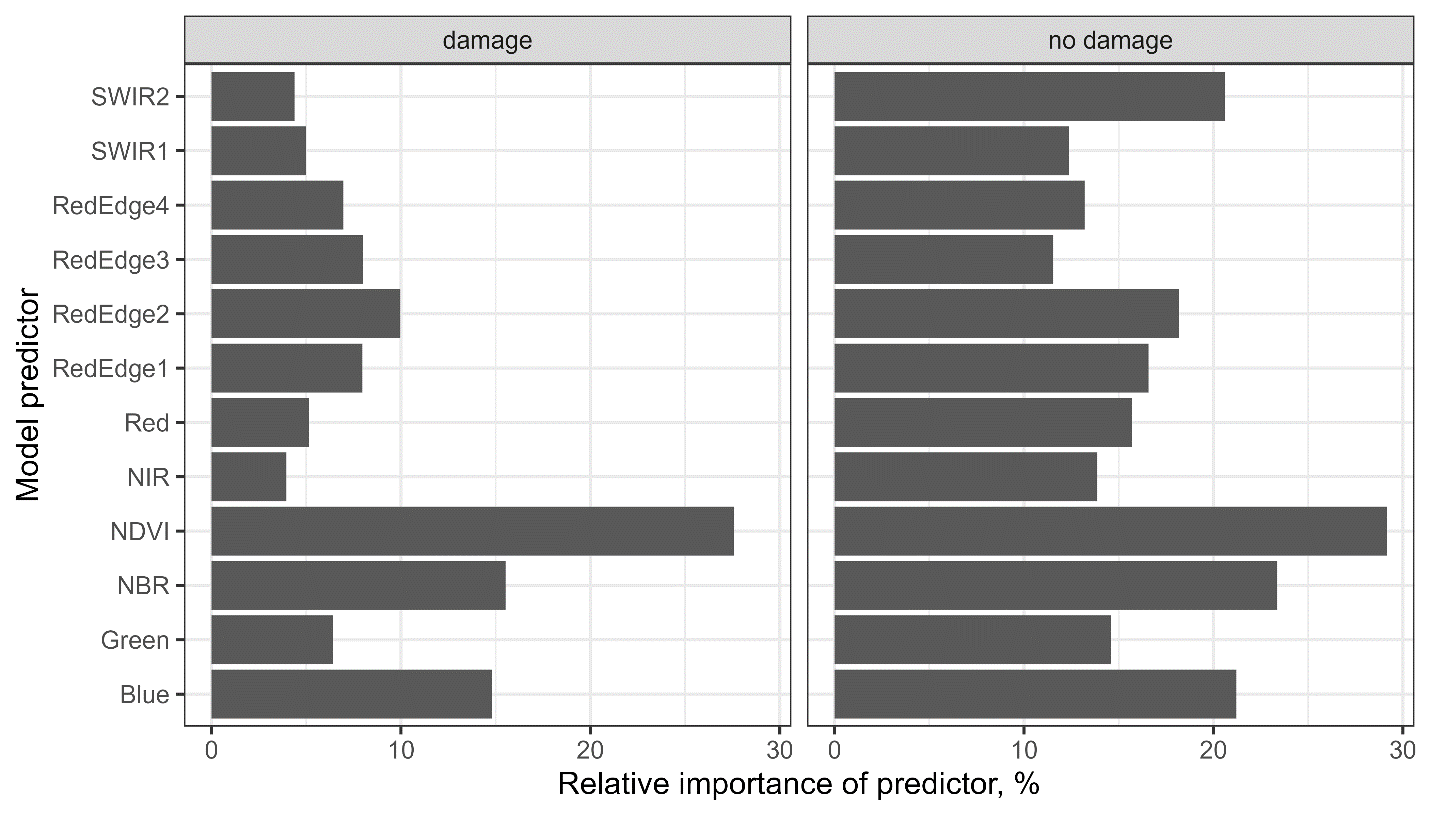


Figure S5. The relative importance of RF damaged forest cover (2022) binary model. Model predictor abbreviatures are as in Figure S2 caption.


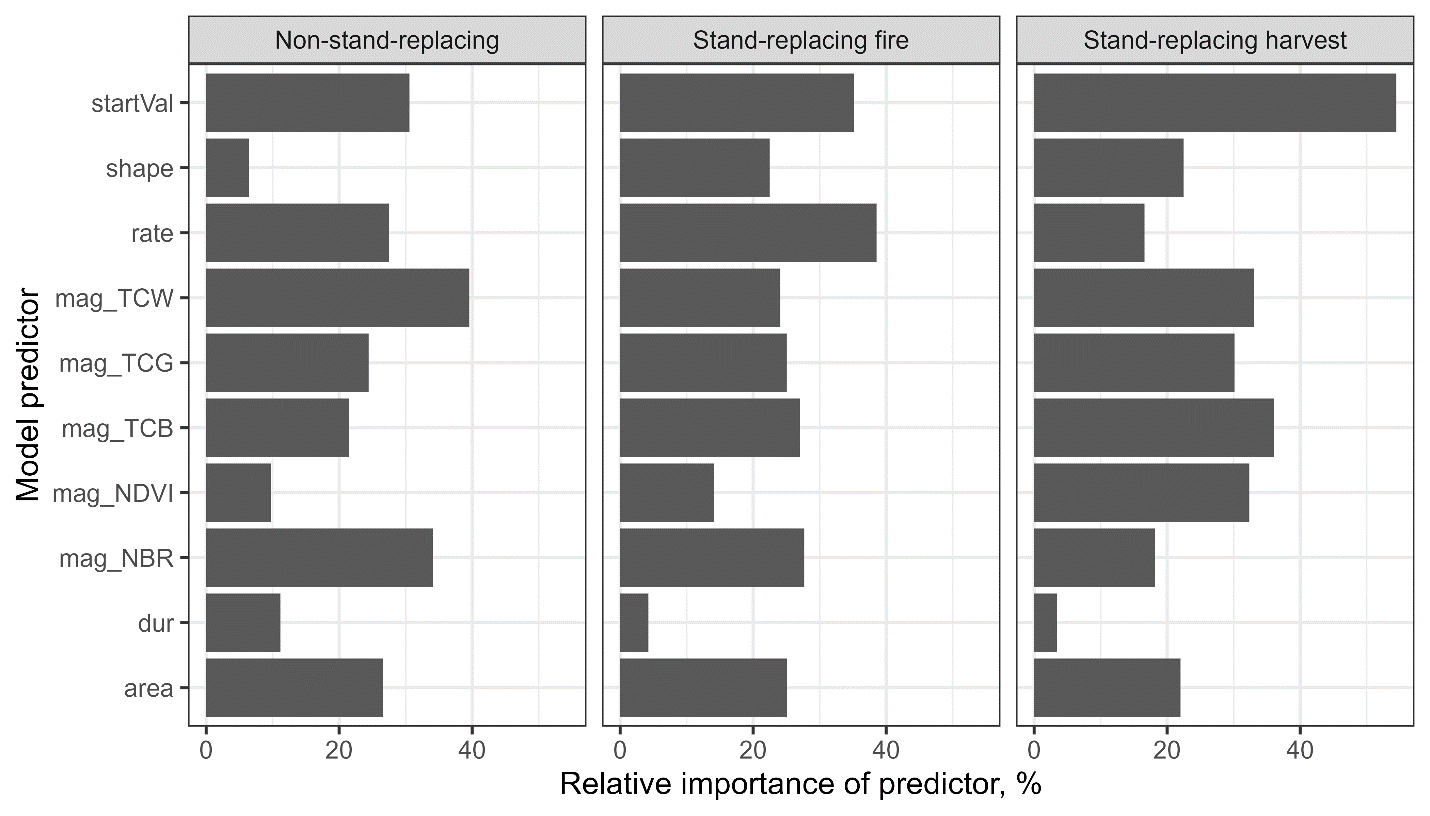


Figure S6. The relative importance of RF pre-war forest disturbance causal agent model. Model predictor abbreviatures: startVal – NBR value before disturbance segment; shape – fractal index of raster patch with adjacent pixels of a same year of disturbance; rate – NBR magnitude divided by segment duration; mag_TCW – magnitude of tasseled cap wetness; mag_TCB – magnitude of tasseled cap brightness; mag_TCG – magnitude of tasseled cap greenness; dur – duration of disturbance segment (years); area – the area of raster patch with adjacent pixels of the same year of disturbance.
