# Supplementary figures and images for: War drives forest fire risks and highlights the need for more ecologically-sound forest management in post-war Ukraine
Source: Sci Rep. 2024 Feb 19;14:4131. doi: 10.1038/s41598-024-54811-5 (PMC10876951; doi:10.1038/s41598-024-54811-5)

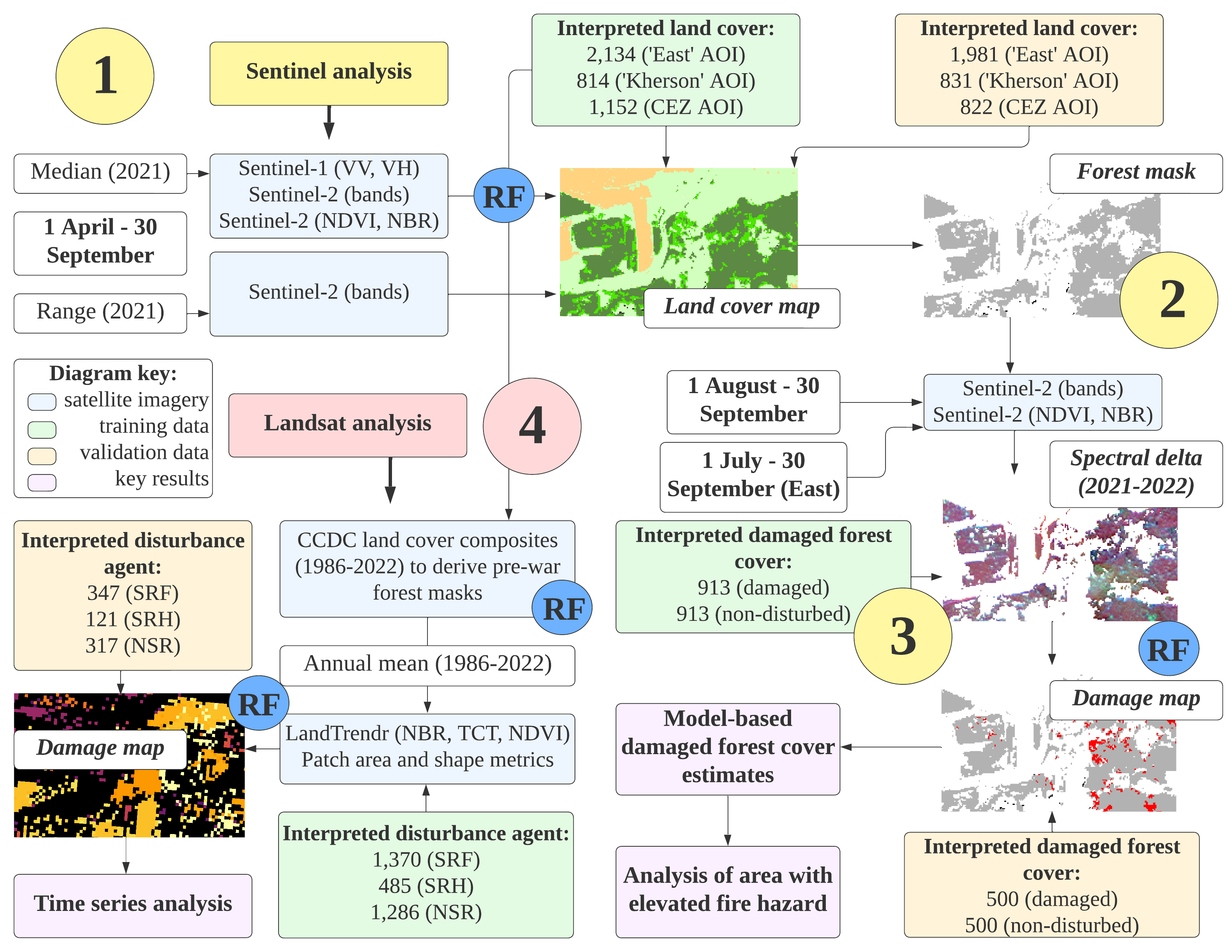

Supplement: Supplementary file 1 — Supplementary Information. [file 41598_2024_54811_MOESM1_ESM.zip › Figure_S1.png]

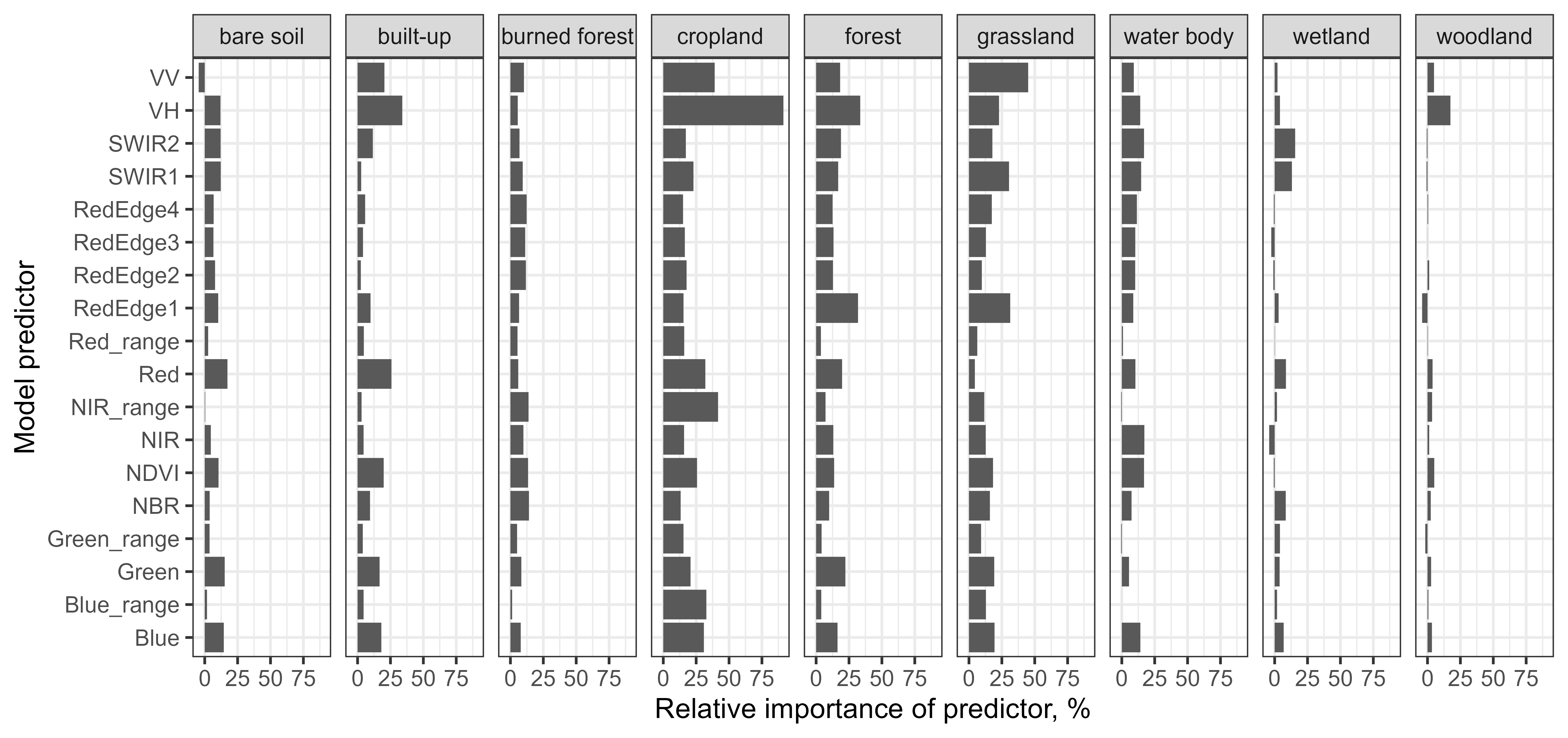

Supplement: Supplementary file 1 — Supplementary Information. [file 41598_2024_54811_MOESM1_ESM.zip › Figure_S2.png]

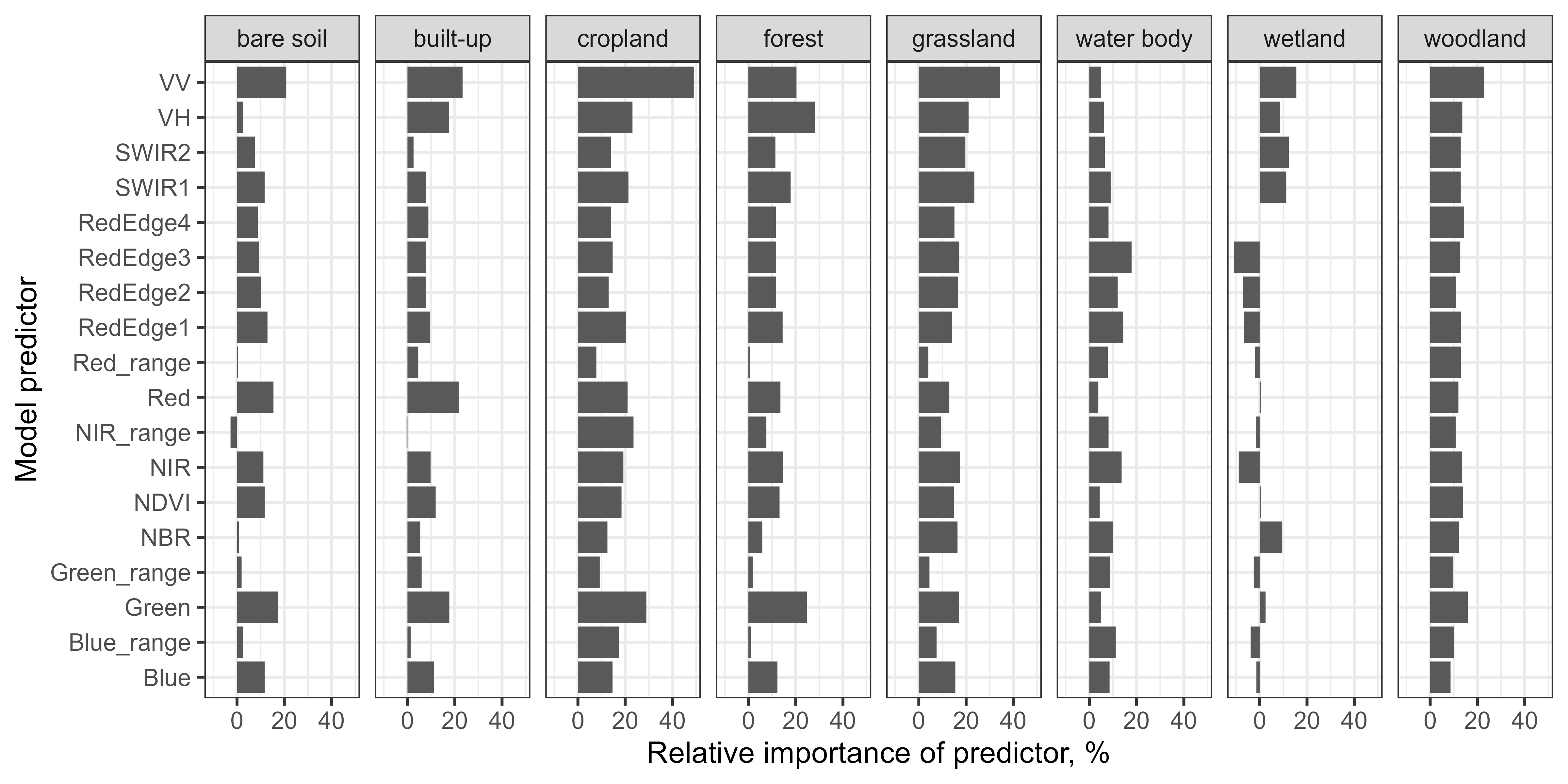

Supplement: Supplementary file 1 — Supplementary Information. [file 41598_2024_54811_MOESM1_ESM.zip › Figure_S3.png]

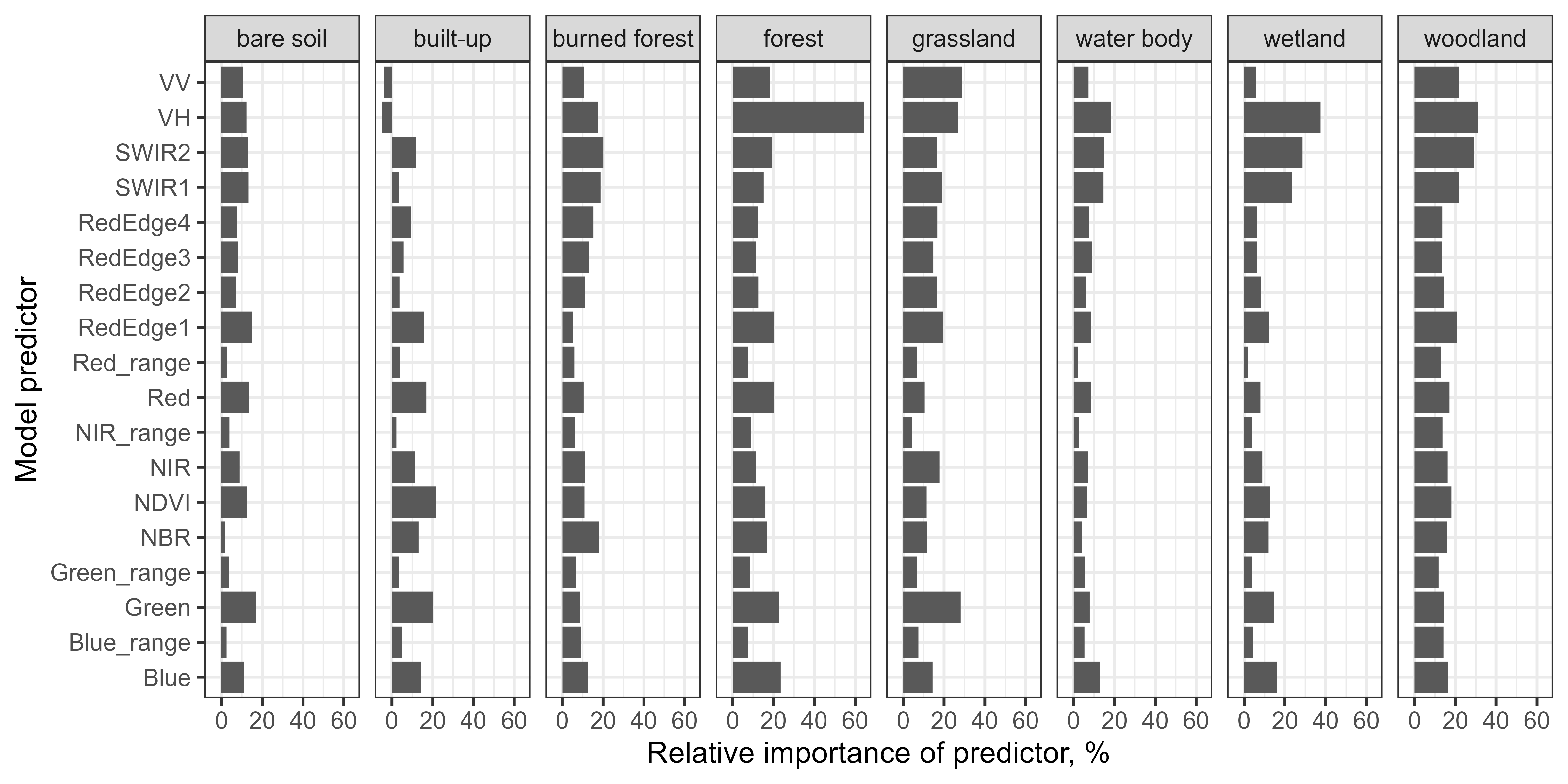

Supplement: Supplementary file 1 — Supplementary Information. [file 41598_2024_54811_MOESM1_ESM.zip › Figure_S4.png]

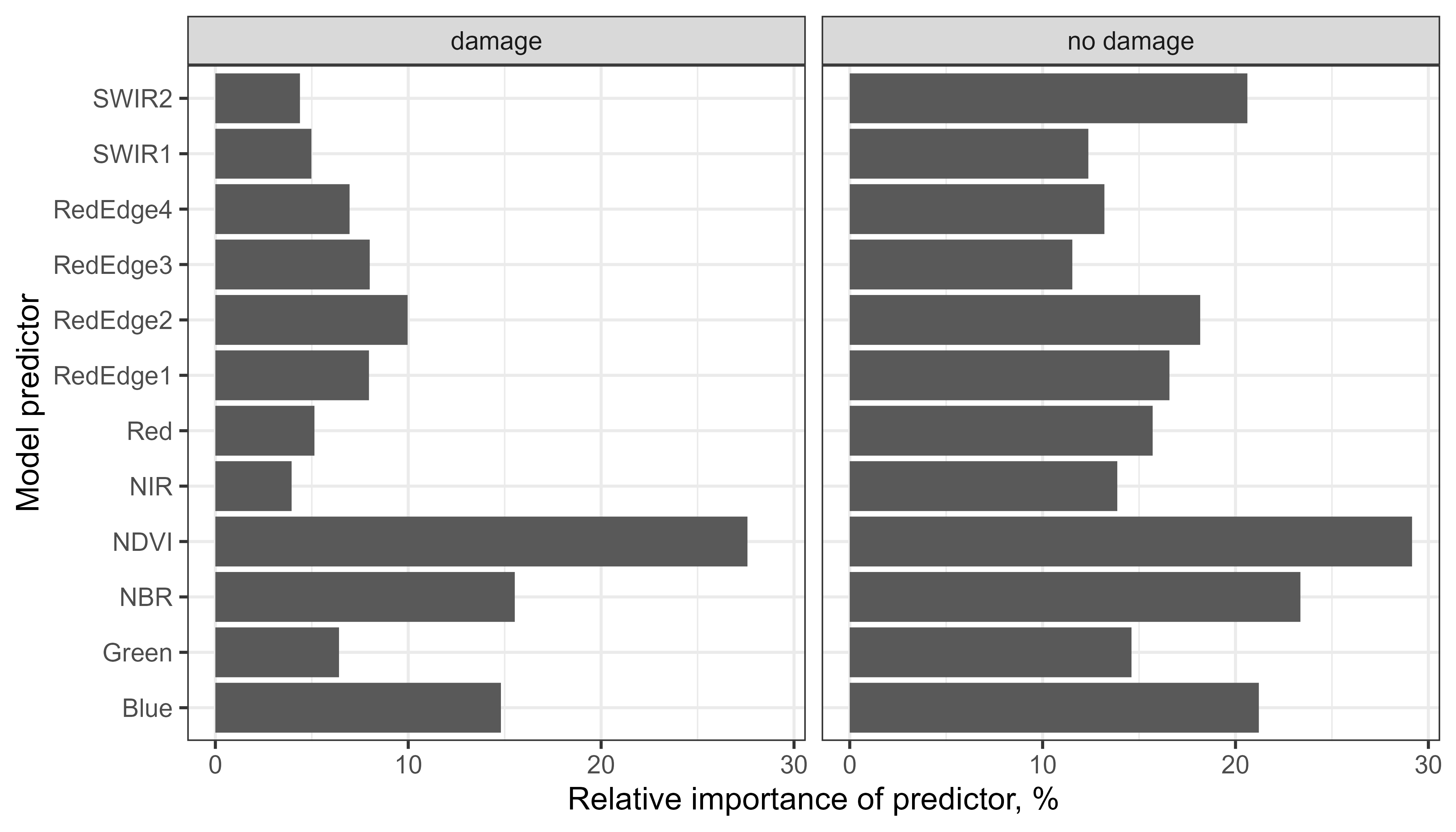

Supplement: Supplementary file 1 — Supplementary Information. [file 41598_2024_54811_MOESM1_ESM.zip › Figure_S5.png]

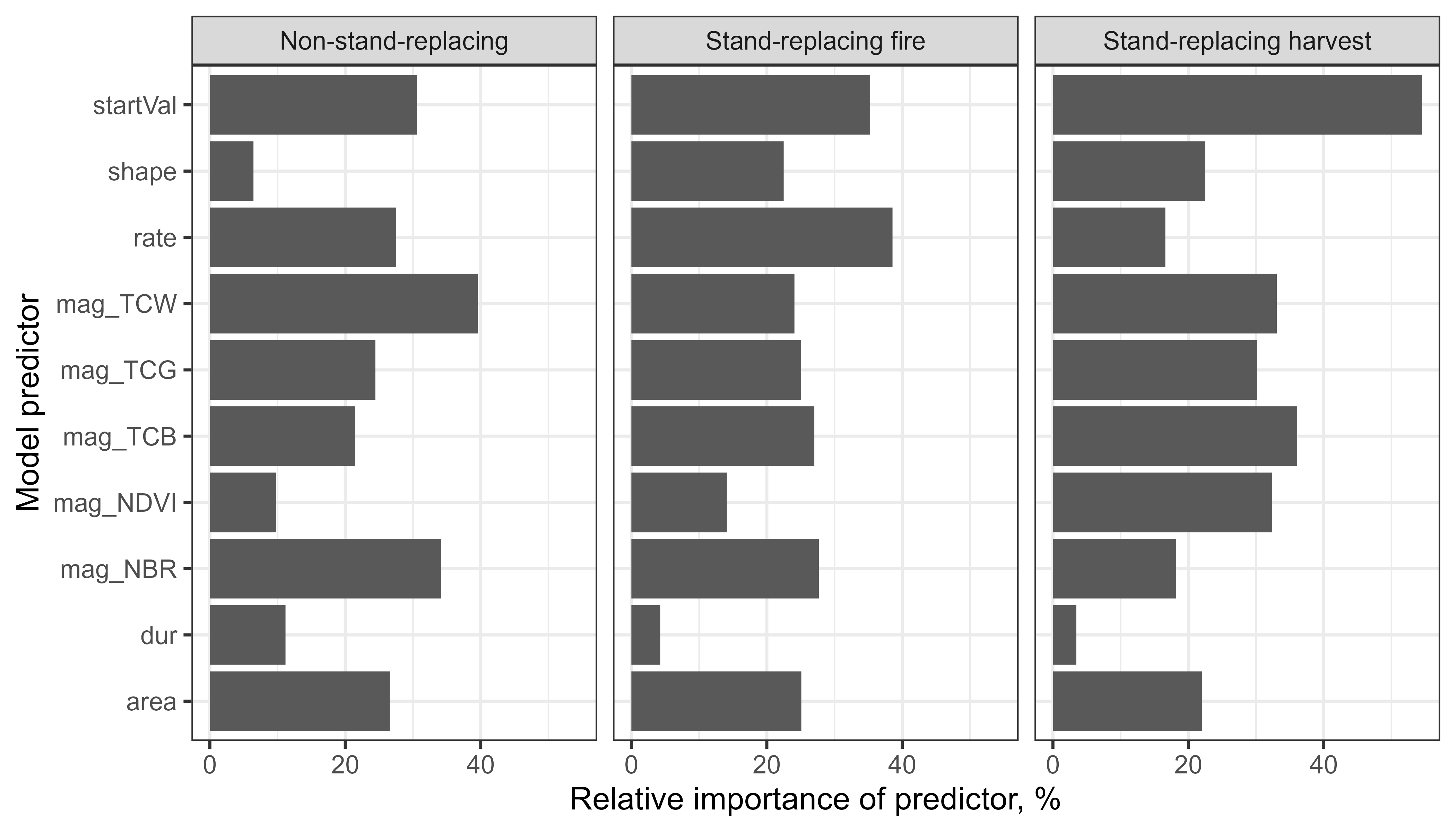

Supplement: Supplementary file 1 — Supplementary Information. [file 41598_2024_54811_MOESM1_ESM.zip › Figure_S6.png]
